# Effects of mind-body exercise in chronic cardiopulmonary dyspnoea patients—a network meta-analysis of randomized controlled trials

**DOI:** 10.3389/fcvm.2025.1546996

**Published:** 2025-06-04

**Authors:** Yilin Li, Jing Wu, Rong Lei

**Affiliations:** ^1^ Department of Geriatrics, The Third People's Hospital of Chengdu, Chengdu, China; ^2^ Health Management Center, Affiliated Hospital of North Sichuan Medical College, Nanchong, Sichuan, China

**Keywords:** exercise interventions, chronic cardiopulmonary dyspnoea, yoga, network meta-analysis, mind-body exercises

## Abstract

**Background:**

The objective of this meta-analysis is to evaluate and compare the effectiveness of various mind-body exercises in the rehabilitation of individuals with chronic cardiopulmonary dyspnoea.

**Design:**

A systematic review and network meta-analysis was conducted on articles from PubMed, EMBASE, Cochrane Library, Web of Science, and China National Knowledge infrastructure which were searched up to November 21, 2024. The methodological quality of the included trials was evaluated using the Cochrane Risk of Bias tool. A network meta-analysis was performed to compare the effects of various mind-body therapies, including Liuzijue, Baduanjin, Yoga, Tai Chi, Wuqinxi, Qigong, Yijinjing, and Buddhist walking meditation.

**Results:**

The analysis included 44 randomized controlled trials involving a total of 2,957 subjects. The network meta-analysis indicated that Yoga might be the most effective exercise intervention for reducing the MLHFQ score (SUCRA: 71.8%). Yoga training demonstrated superior results in increasing the 6-minute walk distance (6MWD) (SUCRA: 99.7%). Qigong was also identified as the most effective exercise intervention for decreasing St. George's Respiratory Questionnaire (SGRQ) (SUCRA: 97.1%). When compared with other treatments, Baduanjin is likely the most appropriate intervention for decreasing COPD Assessment Test (CAT) (SUCRA: 87.1%). Regarding N-terminal pro-B-type natriuretic peptide (NT-proBNP), Yoga might be more advantageous for decreasing the NT-proBNP (SUCRA: 83.4%).

**Conclusion:**

The findings of this study suggest that mind-body exercise may represent a promising intervention for the management of chronic cardiopulmonary dyspnoea. Our findings indicate that Yoga might be the most effective exercise intervention for improving the MLHFQ scores, 6MWD, and NT-proBNP. Qigong is identified as the most effective exercise intervention for decreasing SGRQ. Compared to other treatment methods, Baduanjin may be the most suitable intervention for lowering CAT. This study recommends that patients with chronic cardiopulmonary dyspnoea select appropriate mind-body exercises to achieve effective management of chronic cardiopulmonary dyspnoea.

**Systematic Review Registration:**

https://inplasy.com/inplasy-2024-11-0092/, INPLASY INPLASY2020100052.

## 1 Introduction

### 1.1 Background

Chronic cardiopulmonary dyspnoea is a complex and severe clinical syndrome and a heavy burden for patients with a decline in cardiopulmonary function (1–3). The most prevalent types include chronic obstructive pulmonary disease (COPD), pulmonary fibrosis, pulmonary artery hypertension, pulmonary embolism, heart failure (HF), valvular heart disease, and myocardiopathies (4, 5). Dyspnoea is considered chronic if present for more than 1 month (6, 7). Due to its high incidence and mortality rates, chronic cardiopulmonary dyspnoea has become a significant public health issue (8, 9). With the advancement of modern medicine, exercise rehabilitation has emerged as a core element of cardiopulmonary rehabilitation and has become one of its most important aspects, enabling patients to improve their disease status through active exercise (10–12).

Mind-body exercises primarily include Liuzijue, Baduanjin, Yoga, Tai Chi, Wuqinxi, Qigong, Yijinjing, and Buddhist walking meditation (BWM) (13, 14). Mind-body exercises are gentle, require minimal equipment or external assistance, and are not constrained by location or time, making them easy to perform and learn. Therefore, they are suitable for individuals with compromised health, such as those with cardiopulmonary dyspnoea (15, 16). Particularly, mind-body therapies combine physical exercise with breathing and deep relaxation techniques, which can enhance physical function as well as emotional and mental awareness, and therefore may provide greater benefits than other forms of traditional exercise.

However, different mind-body exercise programs have distinct characteristics and may yield varying effects on patients with chronic cardiopulmonary dyspnoea. Currently, there is a lack of evidence-based recommendations to determine which exercise program is most suitable for patients with chronic cardiopulmonary dyspnoea. Compared to traditional treatment, practicing Tai Chi was effective in alleviating depressive symptoms in HF patients (17, 18). Mind-body exercise could reduce levels of anxiety and depression in those with COPD (19).

Currently, there is no consensus on the best practice plans for different mind-body exercises in the rehabilitation of patients with chronic cardiopulmonary dyspnoea. Therefore, the primary objective of this study is to conduct a network meta-analysis (NMA) of randomized controlled trials (RCTs) to determine the effects of different mind-body exercises, evaluate the impact of these exercises on the Minnesota Living with Heart Failure Questionnaire (MLHFQ), 6-minute walk distance (6MWD), St. George's Respiratory Questionnaire (SGRQ), COPD Assessment Test (CAT), and N-terminal pro-B-type natriuretic peptide (NT-proBNP) in patients with chronic cardiopulmonary dyspnoea, and provide a better understanding of the effects of these mind-body exercises for patients and clinicians.

## 2 Materials and methods

### 2.1 Search strategy

The researchers in this study searched five electronic databases (PubMed, EMBASE, Cochrane Library, Web of Science, and China National Knowledge Infrastructure) from their inception until November 21, 2024. The detailed search strategy can be found in Supplementary Table S1. INPLASY registration number: INPLASY2020100052.

### 2.2 Inclusion criteria

(1) Experimental group utilizing different mind-body exercise methods as interventions for cardiopulmonary dyspnoea; (2) Control group receiving only standard care and patient rehabilitation; (3) Clinical randomized controlled trials; (4) Outcomes must include at least one of the following indicators: MLHFQ, 6MWD, SGRQ, CAT, and NT-proBNP.

### 2.3 Exclusion criteria

(1) Studies with incomplete or unreported data; (2) Studies from non-randomized controlled trials [including quasi-randomized controlled trials, letters to editors, case reports, reviews, animal studies, protocols, or conference abstracts]; (3) No language restrictions exist.

### 2.4 Outcomes

The MLHFQ is a health-related quality of life (HRQoL) questionnaire specifically for patients with this heart condition, with potential value in both research and clinical practice (20). Scores range from 0 to 105, with lower scores reflecting better health-related quality of life (21). This questionnaire is effective and reliable for assessing quality of life (QOL) in patients with HF (22) (Supplementary Table S2). The 6MWD test was used to assess physical functional capacity. Patients were required to walk back and forth in a corridor with a length of 30 m as soon as possible, and the test was terminated at 6 min, and the walking distance was measured (23). The longer the distance, the better the exercise endurance of patients. The SGRQ was selected as the primary outcome due to its validated use in COPD trials (Supplementary Table S3). This disease-specific instrument assesses health-related quality of life (HRQoL) through three domains (symptoms, impact, and activity) and a total score (0–100), where higher scores indicate worse health status (24). Extensively validated in Western and Chinese COPD populations, the SGRQ demonstrates strong reliability, sensitivity, and responsiveness to clinical change, making it ideal for evaluating treatment effects (25–28). The CAT is an 8-item patient-reported questionnaire assessing COPD impact on quality of life (29) (Supplementary Table S4). Patients score each item from 0 (no impairment) to 5 (severe impairment) (30). Total scores range from 0 to 40, with higher scores indicating worse symptoms and poorer health status. Normal range of NTproBNP is <125 pg/ml (31). The minimal clinically important difference (MCID) for MLHFQ ranged from 3.59 to 19.14 points (32). The MCID for the 6MWD is 26 m (33). Mean and range of MCID estimates for SGRQ is 3.9 (0.7–5.5) (34). The estimated MCID of the CAT for the improvement directions was at least −3 points (35). The MCID for the NT-proBNP is a reduction of more than 30% (36).

### 2.5 Study selection

The literature was screened and excluded using the reference management software EndNote. Two researchers initially screened the titles of duplicate literature, non-randomized controlled trials, review articles, conference papers, protocols, and letters. The same two researchers then reviewed the abstracts to determine which literature to include and which to exclude. Finally, the researchers conducted a full reading of the remaining literature and made further determinations for inclusion. Throughout this process, the two researchers independently screened the literature and compared the remaining studies; if they agreed, the studies were included, and if they disagreed, a third researcher discussed and resolved the differences.

### 2.6 Data extraction

A five-item, standardised and pre-selected data extraction form was used to record data for inclusion in the study under the following headings: (1) author, (2) country, (3) year of publication, (4) sample size, and (5) details of the exercise intervention.

### 2.7 Risk of bias of individual studies

Two researchers independently assessed the risk of bias in randomized controlled trials using the Cochrane Handbook version 5.1.0. The following seven domains were considered: (1) random sequence generation, (2) allocation concealment, (3) blinding of participants, (4) blinding of personnel, (5) incomplete outcome data, (6) selective reporting, and (7) other sources of bias. Trials were categorized into three risk levels based on the number of components with a potential high risk: high risk, unclear risk, and low risk. Based on the number of components with a potential high risk ratio, trials were classified into three risk ratio levels: high risk (five or more), moderate risk (three or four), and low risk (two or fewer) (37).

### 2.8 Data analysis

In studies where exercise is used as an intervention, all variables are continuous and are reported as mean values with standard deviation (SD) (38). Continuous variables in the study will be reported with 95% confidence intervals (CIs) and analyzed as mean differences (MDs), defined as the absolute difference between the means of the treatment and control groups, calculated using the same scale. Given the potential heterogeneity among different studies, we opted for a random effects model for the analysis rather than a fixed effects model (39). Egger's test, Begg's test, and funnel plots were used to test for publication bias.

We used Stata software (version 15.1) and, following the PRISMA NMA guidelines, conducted network meta-analysis aggregation and analysis using Markov Chain Monte Carlo simulations within a Bayesian framework (40). If the *P*-value > 0.05, we will use the node-splitting method to quantify and demonstrate the consistency between indirect and direct comparisons calculated using commands in Stata software. The consistency test was passed (41).

Stata software was used to present and describe network diagrams of different exercise interventions. In the generated network diagrams, each node represents different exercise interventions and various control conditions, while the lines connecting the nodes represent direct comparisons between interventions. The size of each node and the width of the connecting lines are proportional to the number of studies (42).

The levels of intervention were summarized and reported as *P*-values. *P*-values are considered as frequency simulation values under the cumulative ranking curve (SUCRA), which measure the degree of certainty that one treatment is superior to another and represent the average of all competing treatments. *P*-values range from 0 to 1, where 1 indicates the best treatment with no uncertainty, and 0 indicates the worst treatment with no uncertainty. Although *P*-scores or SUCRA can be effectively reinterpreted as percentages of effectiveness or acceptability for exercise interventions, such scores should be interpreted cautiously unless there are meaningful clinical differences between the interventions (43). To assess whether there is bias in small-scale studies (which may lead to publication bias in NMA), a network funnel plot was generated and visually inspected using symmetry criteria (44).

## 3 Results

### 3.1 Study and identification and selection

**Figure 1:**
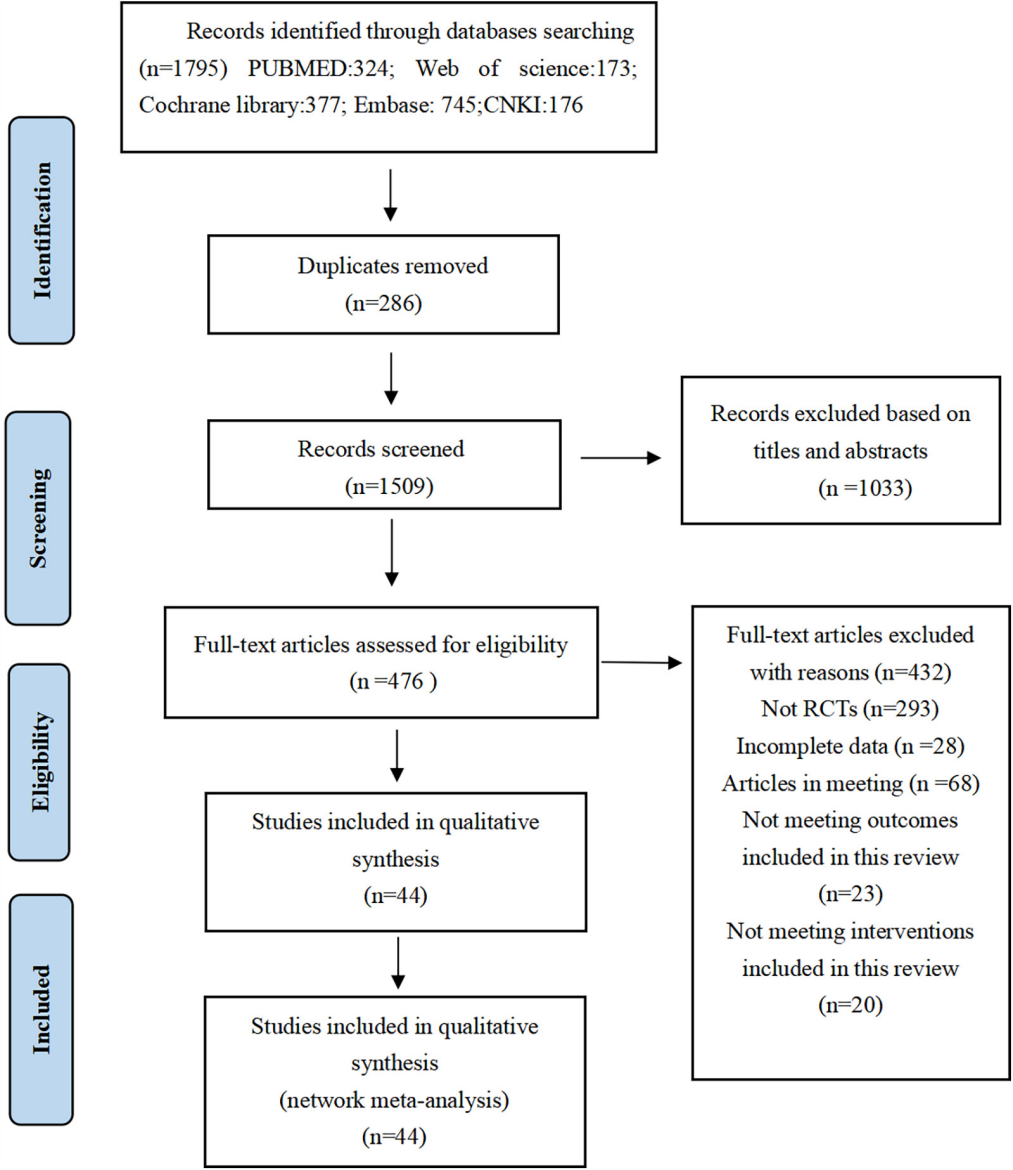
Flow diagram of study inclusion.

A total of 1,795 documents were retrieved from electronic databases. After removing duplicates, the remaining 1,509 documents had their titles and abstracts reviewed, resulting in the exclusion of 1,033 documents. The remaining 476 documents were read in full, and 432 documents were excluded for reasons including, but not limited to, the following: non-randomized controlled trials, incomplete data, conference papers, and interventions that did not meet the criteria for this review. Ultimately, 44 documents were included in this study (Figure 1).

### 3.2 Quality assessment of the included studies

Nineteen studies were defined as low risk, four as high risk, and 21 as moderate risk. None of these studies achieved blinding of both participants and assessors, but it was difficult to blind both participants and assessors due to the nature of the interventions, which involved exercise, as patients and their families were required to sign informed consent forms before the start of the trial. Specific details will be presented in Supplementary Table S5, Supplementary Figure S1, Supplementary Figure S2.

### 3.3 Characteristics of the included studies

In summary, we included 44 randomized controlled trials, involving a total of 2,957 patients diagnosed with chronic cardiopulmonary dyspnoea (16, 45–89). The interventions in the treatment group included Liuzijue (6 studies), Baduanjin (11 studies), BWM (1 studies), Qigong (4 studies), Tai Chi (15 studies), Wuqinxi (1 studies), Yijinjing (1 studies), and Yoga (5 studies). Eleven studies reported MLHFQ as an outcome measure, 32 studies reported MLHFQ, 2 studies reported CAT, 4 studies reported SGRQ, and 6 studies reported NT-proBNP. Of the 44 studies, 7 was conducted in USA, 1 in the UK, 1 in Thailand, 2 in Sweden, 1 in the Italy, 2 in India, and the rest were from different regions in China. The follow-up durations of the studies ranged from 1 months to 6 months. Table 1 presents the characteristics of the included studies.

**Table 1:** Characteristics of the studies included in the meta-analysis.

Author	Country	Year	Diagnosis	Sample Size (T)	Sample Size (C)	Intervention	Control	Outcome	Follow up (months)	Frequency (times/week)	Exercise duration (minutes)
L Zheng	China	2017	HF	9	8	Liuzijue	CON	6MWD MLHFQ NT-proBNP	≤3	≥5	<50
Z Zhu	China	2010	HF	20	22	Liuzijue	CON	6MWD	≤3	≥5	<50
M Xu	China	2019	HF	40	40	Baduanjin	CON	6MWD MLHFQ	>3	≥5	<50
H Ewa	Sweden	2017	HF	18	12	Yoga	CON	6MWD NT-proBNP	≤3	<5	≥50
GY Yeh	USA	2013	HF	8	8	Tai Chi	CON	6MWD MLHFQ	≤3	<5	≥50
LS Redwine	USA	2019	HF	25	22	Tai Chi	CON	6MWD	>3	<5	≥50
C Giuseppe	Italy	2011	HF	30	30	Tai Chi	CON	6MWD NT-proBNP	≤3	<5	≥50
C Huang	China	2014	HF	44	44	Tai Chi	CON	6MWD MLHFQ	≤3	≥5	<50
ML Yu	China	2018	HF	54	55	Baduanjin	CON	6MWD MLHFQ NT-proBNP	≤3	<5	<50
MI Polkey	UK	2018	COPD	55	55	Tai Chi	CON	6MWD SGRQ	≤3	≥5	≥50
AWK Chan	China	2013	COPD	70	69	Tai Chi	CON	6MWD SGRQ	>3	<5	≥50
ST Du	China	2012	COPD	36	38	Tai Chi	CON	6MWD CAT SGRQ	≤3	<5	≥50
ZF Ren	China	2017	COPD	30	30	Tai Chi	CON	6MWD CAT	≤3	<5	≥50
Y Zhang	China	2019	COPD	50	50	Tai Chi	CON	6MWD CAT	>3	≥5	≥50
XC Zhang	China	2014	COPD	18	18	Tai Chi	CON	6MWD CAT	>3	≥5	≥50
M Ding	China	2015	COPD	10	10	Baduanjin	CON	6MWD SGRQ	≤3	<5	<50
Y Zhu	China	2010	COPD	26	27	Wuqinxi	CON	6MWD	≤3	≥5	<50
XS Dong	China	2021	COPD	10	10	Qigong	CON	6MWD CAT SGRQ	≤3	<5	<50
J Zhang	China	2017	COPD	50	50	Liuzijue	CON	SGRQ	≤3	≥5	<50
DP fang	China	2012	COPD	61	60	Liuzijue	CON	6MWD	>3	≥5	<50
JX Chen	China	2009	COPD	31	29	Liuzijue	CON	6MWD SGRQ	≤3	≥5	<50
Q Chen	China	2021	COPD	49	49	Qigong	CON	6MWD CAT	>3	≥5	<50
CM Xiao	China	2015	COPD	59	60	Liuzijue	CON	6MWD	>3	<5	<50
XD Liu	China	2012	COPD	51	32	Qigong	CON	6MWD	>3	<5	≥50
Q Li	China	2012	COPD	30	30	Tai Chi	CON	SGRQ	>3	≥5	≥50
SM Chen	China	2020	COPD	60	60	Baduanjin	CON	CAT	>3	≥5	<50
YC Zheng	China	2019	COPD	18	14	Baduanjin	CON	6MWD CAT	≤3	≥5	<50
DF Hou	China	2017	COPD	25	23	Baduanjin	CON	6MWD	≤3	<5	<50
G Yin	China	2013	COPD	10	12	Baduanjin	CON	6MWD SGRQ	>3	≥5	<50
Y Gao	China	2015	COPD	55	57	Yijinjing	CON	6MWD CAT	>3	≥5	≥50
BHP Ng	China	2011	COPD	23	29	Baduanjin	CON	6MWD	>3	≥5	<50
L Ng	China	2014	COPD	68	70	Tai Chi	CON	6MWD SGRQ	>3	<5	≥50
HJ Liu	China	2021	COPD	40	40	Baduanjin	CON	CAT	>3	≥5	<50
M Zhou	China	2021	IPF	32	31	Qigong	CON	6MWD SGRQ	>3	≥5	≥50
Jain AK	India	2022	HF	30	30	Yoga	CON	MLHFQ NT-proBNP	≤3	≥5	≥50
Yeh GY	USA	2011	HF	50	50	Tai Chi	CON	MLHFQ 6MWD	≤3	<5	≥50
Yeh GY	USA	2008	HF	15	15	Tai Chi	CON	MLHFQ 6MWD	≤3	<5	≥50
Donesky D	USA	2017	HF who also had COPD	7	8	Yoga	CON	6MWD	≤3	<5	≥50
Pullen PR	USA	2008	HF	9	10	Yoga	CON	MLHFQ	≤3	<5	≥50
Yeh GY	USA	2004	HF	15	15	Tai Chi	CON	MLHFQ 6MWD	≤3	<5	≥50
Ma CH	China	2022	HF	68	68	Baduanjin	CON	6MWD	>3	<5	<50
Chen XK	Sweden	2021	HF	8	10	Baduanjin	CON	MLHFQ NT-proBNP	≤3	≥5	<50
Krishna BH	India	2014	HF	44	48	Yoga	CON	MLHFQ 6MWD	≤3	≥5	≥50
Srisoongnern S	Thailand	2021	HF	24	24	BWM	CON	MLHFQ 6MWD	≤3	<5	<50

CON, control group with routine care (no exercise); T, experimental group; C, control group; HF, heart failure; COPD, chronic obstructive pulmonary disease; IPF, idiopathic pulmonary fibrosis; MLHFQ, Minnesota living with heart failure questionnaire; 6MWD, 6-minute walk distance; SGRQ, St. George's respiratory questionnaire; CAT, chronic obstructive pulmonary disease (COPD) assessment test; BWM, Buddhist walking meditation; NT-proBNP, N-terminal pro-B-type natriuretic peptide.

### 3.4 Network meta-analysis

The full NMA figure will be shown in Supplementary Figures S3A–S3E.

#### 3.4.1 Minnesota living with heart failure questionnaire (MLHFQ)

**Figure 2:**
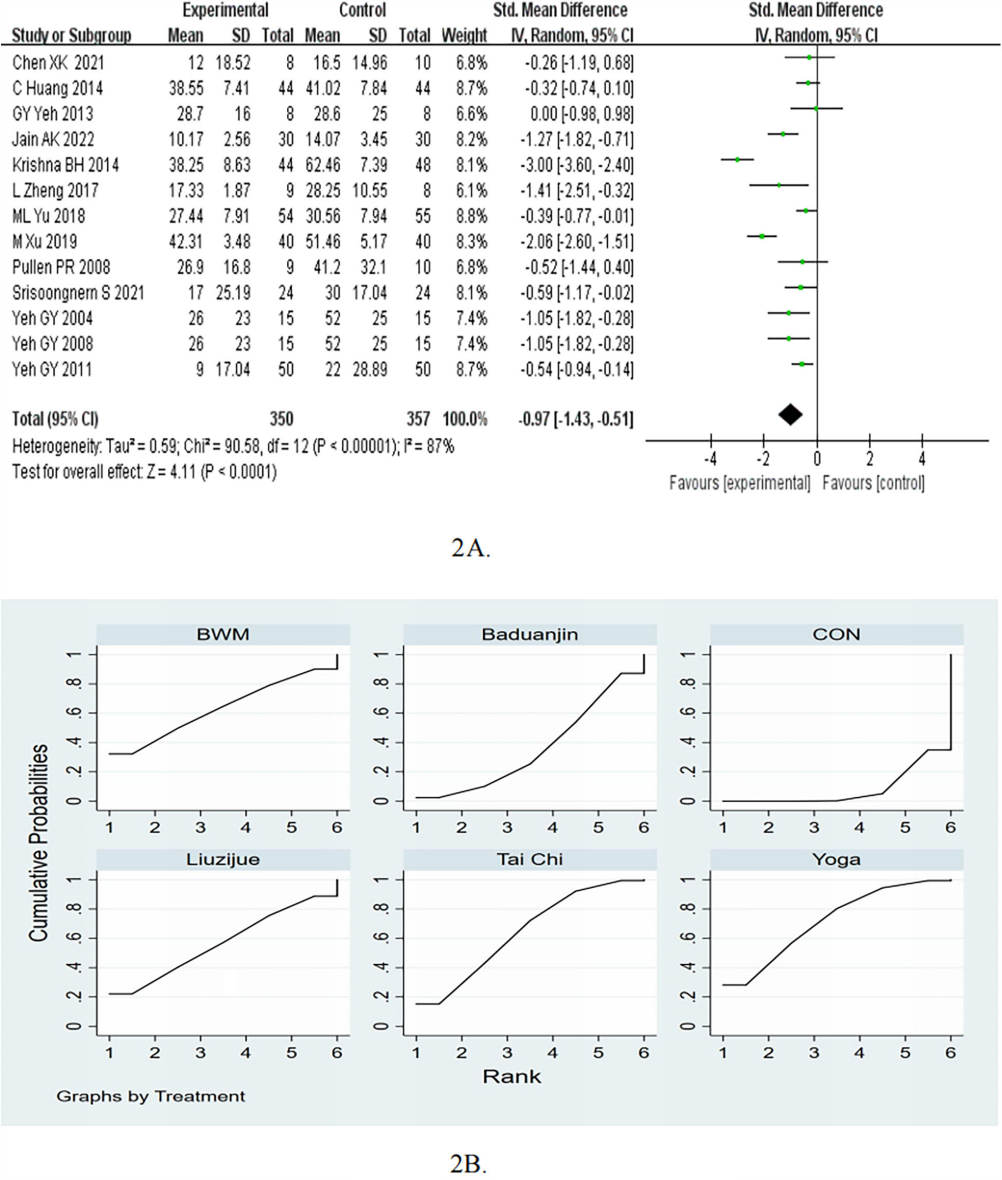
**(A)** Forest plots for the effects of mind-body exercise on MLHFQ. **(B)** SUCRA plot for MLHFQ.

Thirteen studies examined the effects of mind-body exercise on the MLHFQ. The meta-analysis shown that mind-body exercise was effective in reduce the MLHFQ [standardised mean difference (SMD) = −0.97, 95%CI −1.43 to −0.51, *p* < 0.05, *I*^2^ = 87%; Figure 2A], as compared to control groups.

In the subgroup of follow up, the mind-body exercise showed a significant improvement in MLHFQ compared to the control group for ≤3months (pooled SMD: −0.87, 95% CI: −1.32 to −0.42, *p* < 0.05) and >3months (pooled SMD: −2.06, 95% CI: −2.60 to −1.51, *p* < 0.05), as shown in Table 2. In the subgroup of frequency, the mind-body exercise showed a significant improvement in MLHFQ compared to the control group for ≥5 times/week (pooled SMD: −1.40, 95% CI: −2.30 to −0.49, *p* < 0.05) and <5 times/week (pooled SMD: −0.55, 95% CI: −0.77 to −0.34, *p* < 0.05). In the subgroup of exercise duration, the mind-body exercise showed a significant improvement in MLHFQ compared to the control group for ≥50 min (pooled SMD: −1.09, 95% CI: −1.83 to −0.35, *p* < 0.05) and <50 min (pooled SMD: −0.82, 95% CI: −1.41 to −0.22, *p* < 0.05).

**Table 2:** Subgroup meta-analysis of mind-body exercise for chronic cardiopulmonary dyspnoea.

Categories	Outcomes	Subgroup	Pooled SMD(95%CI)	*P* value	Heterogeneity	
					*I* ^2^	*P* value
Follow up	MLHFQ	Overall	−0.97 [−1.43, −0.51]	<0.05	87%	<0.05
		≤3 months	−0.87 [−1.32, −0.42]	<0.05	85%	<0.05
		>3 months	−2.06 [−2.60, −1.51]	<0.05	NA	NA
	6MWD	Overall	0.53 [0.35, 0.71]	<0.05	78%	<0.05
		≤3 months	0.53 [0.30, 0.75]	<0.05	68%	<0.05
		>3 months	0.53 [0.23, 0.83]	<0.05	85%	<0.05
	SGRQ	Overall	−0.35 [−0.68, −0.01]	<0.05	80%	<0.05
		≤3 months	−0.32 [−0.52, −0.12]	<0.05	0	0.42
		>3 months	−0.43 [−1.14, 0.27]	0.23	91%	<0.05
	CAT	Overall	−0.81 [−1.18, −0.43]	<0.05	82%	<0.05
		≤3 months	−0.88 [−1.77, 0.00]	0.05	86%	<0.05
		>3 months	−0.74 [−1.14, −0.34]	<0.05	80%	<0.05
Frequency	MLHFQ	Overall	−0.97 [−1.43, −0.51]	<0.05	87%	<0.05
		≥5 times/week	−1.40 [−2.30, −0.49]	<0.05	92%	<0.05
		<5 times/week	−0.55 [−0.77, −0.34]	<0.05	0	0.52
	6MWD	Overall	0.53 [0.35, 0.71]	<0.05	78%	<0.05
		≥5 times/week	0.72 [0.42, 1.02]	<0.05	81%	<0.05
		<5 times/week	0.38 [0.17, 0.59]	<0.05	71%	<0.05
	SGRQ	Overall	−0.35 [−0.68, −0.01]	<0.05	80%	<0.05
		≥5 times/week	−0.48 [−1.06, 0.09]	0.1	87%	<0.05
		<5 times/week	−0.19 [−0.53, 0.15]	0.28	58%	0.05
	CAT	Overall	−0.81 [−1.18, −0.43]	<0.05	82%	<0.05
		≥5 times/week	−0.77 [−1.13, −0.41]	<0.05	76%	<0.05
		<5 times/week	−0.82 [−2.03, 0.40]	0.19	90%	<0.05
	NT-proBNP	Overall	−0.46 [−0.93, 0.02]	0.06	71%	<0.05
		≥5 times/week	−0.65 [−1.64, 0.34]	0.2	78%	<0.05
		<5 times/week	−0.23 [−0.51, 0.05]	0.11	0	0.42
Exercise duration	MLHFQ	Overall	−0.97 [−1.43, −0.51]	<0.05	87	<0.05
		≥50 min	−1.09 [−1.83, −0.35]	<0.05	89	<0.05
		<50 min	−0.82 [−1.41, −0.22]	<0.05	84	<0.05
	6MWD	Overall	0.53 [0.35, 0.71]	<0.05	78%	<0.05
		≥50 min	0.41 [0.16, 0.65]	<0.05	76%	<0.05
		<50 min	0.66 [0.39, 0.93]	<0.05	80%	<0.05
	SGRQ	Overall	−0.35 [−0.68, −0.01]	<0.05	80	<0.05
		≥50 min	−0.52 [−1.06, 0.02]	0.06	90	<0.05
		<50 min	−0.16 [−0.42, 0.10]	0.24	0	0.24
	CAT	Overall	−0.81 [−1.18, −0.43]	<0.05	82	<0.05
		≥50 min	−0.92 [−1.48, −0.36]	<0.05	84	<0.05
		<50 min	−0.68 [−1.25, −0.11]	<0.05	83	<0.05
	NT-proBNP	Overall	−0.46 [−0.93, 0.02]	0.06	71	<0.05
		≥50 min	−0.75 [−1.49, −0.01]	<0.05	78	0.01
		<50 min	−0.10 [−0.43, 0.23]	0.55	0	0.97

MLHFQ, Minnesota living with heart failure questionnaire; 6MWD, 6-minute walk distance; SGRQ, St. George's respiratory questionnaire; CAT, chronic obstructive pulmonary disease (COPD) assessment test; NT-proBNP, N-terminal pro-B-type natriuretic peptide; SMD, standardised mean difference; Cl, confidence interval; NA, not available.

Consistency and inconsistency tests were conducted on all *P*-values for indirect and direct comparisons between the studies, with all *P*-values greater than 0.05, indicating that the influence of consistency among the studies is acceptable. Details will be listed in Supplementary Table S6.

The results of the network meta-analysis indicate that yoga [MD = −13.98, 95% CI = (−25.27,−2.69)] and Tai Chi [MD = −12.19, 95% CI = (−21.99,−2.39)] were more effective than control group in reducing the MLHFQ scores. Based on the SUCRA rankings, Yoga had the highest probability of being the most effective therapy for reducing MLHFQ scores, followed by Tai Chi, BWM, Liuzijue, Baduanjin, and control (Table 3A; Figure 2B). Compared to the control group, Yoga showed a decrease of −13.98 (95% CI −25.27 to −2.69) and reach the MCID.

**Table 3A:** League table on MLHFQ.

Yoga	Tai Chi	BWM	Liuzijue	Baduanjin	CON
**Yoga**	1.79 (−13.14, 16.72)	1.14 (−22.63, 24.90)	3.05 (−18.81, 24.92)	8.17 (−7.50, 23.85)	**13.98** (**2.69, 25.27)**
−1.79 (−16.72, 13.14)	**Tai Chi**	−0.65 (−23.75, 22.45)	1.27 (−19.87, 22.40)	6.39 (−8.27, 21.04)	**12.19** (**2.39, 21.99)**
−1.14 (−24.90, 22.63)	0.65 (−22.45, 23.75)	**BWM**	1.92 (−26.15, 29.99)	7.04 (−16.53, 30.60)	12.84 (−8.08, 33.76)
−3.05 (−24.92, 18.81)	−1.27 (−22.40, 19.87)	−1.92 (−29.99, 26.15)	**Liuzijue**	5.12 (−16.53, 26.77)	10.92 (−7.80, 29.65)
−8.17 (−23.85, 7.50)	−6.39 (−21.04, 8.27)	−7.04 (−30.60, 16.53)	−5.12 (−26.77, 16.53)	**Baduanjin**	5.80 (−5.07, 16.68)
**−13.98** (**−25.27, −2.69)**	**−12.19** (**−21.99, −2.39)**	−12.84 (−33.76, 8.08)	−10.92 (−29.65, 7.80)	−5.80 (−16.68, 5.07)	**CON**

Effect sizes where statistical differences exist are bolded.

#### 3.4.2 6-minute walk distance (6MWD)

**Figure 3:**
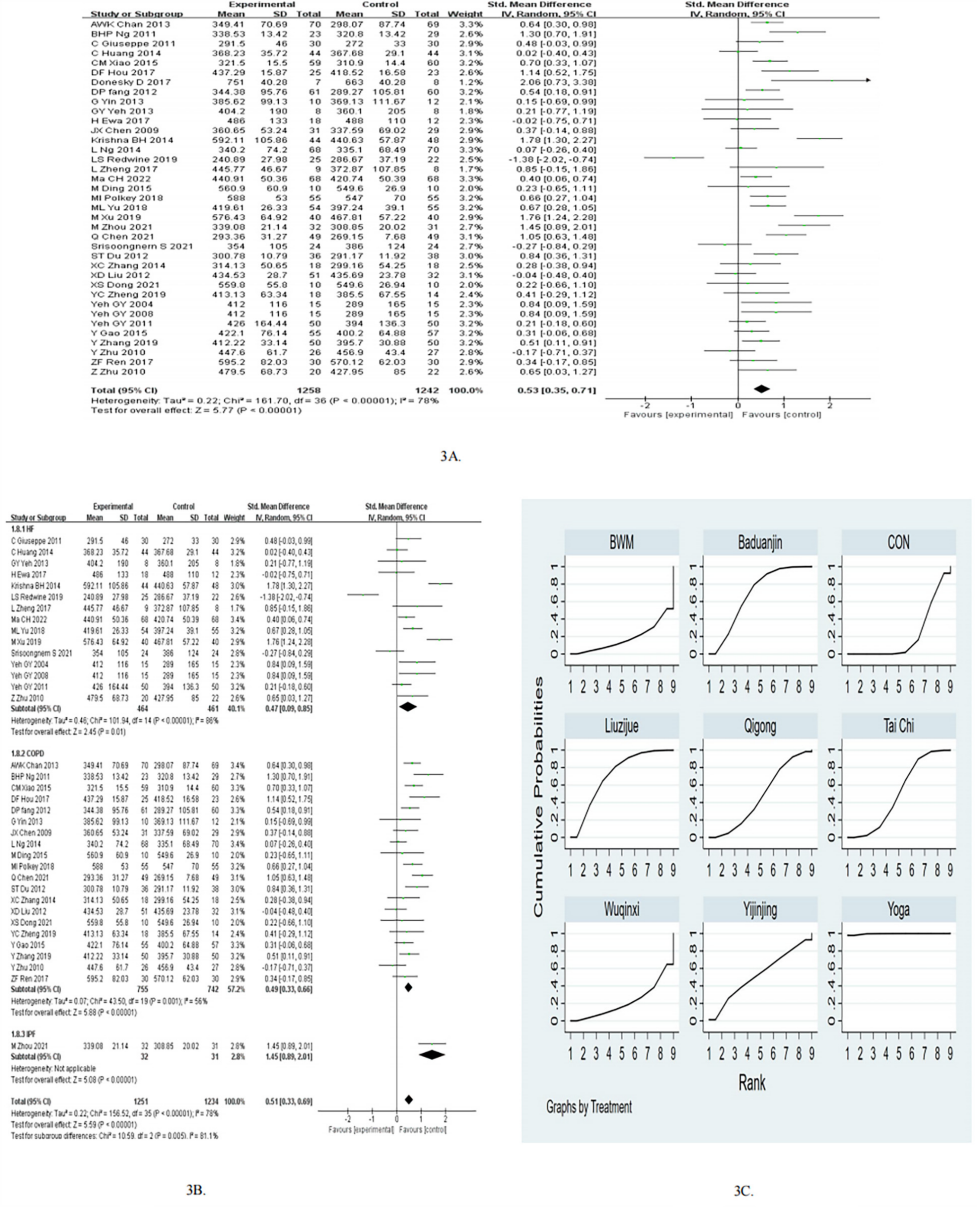
**(A)** Forest plots for the effects of mind-body exercise on 6MWD. **(B)** Subgroup meta-analysis of 6MWD. **(C)** SUCRA plot for 6MWD.

A meta-analysis of the 37 RCTs showed that mind-body exercise was effective in improving 6MWD (SMD = 0.53, 95%CI 0.35–0.71, *p* < 0.05, *I*^2^ = 78%; Figure 3A), as compared to control groups. Subgroup analyses for 6MWD were performed according to different diseases. In the subgroup of HF, the mind-body exercise showed a significant improvement in 6MWD compared to the control group (pooled SMD: 0.47, 95% CI: 0.09–0.85, *p* = 0.01), as shown in Figure 3B. In the subgroup of COPD, the mind-body exercise showed a significant improvement in 6MWD compared to the control group (pooled SMD: 0.49, 95% CI: 0.33–0.66, *p* < 0.01), as shown in Figure 3B.

In the subgroup of follow up, the mind-body exercise showed a significant improvement in 6MWD compared to the control group for ≤3months (pooled SMD: 0.53, 95% CI: 0.30–0.75, *p* < 0.05) and >3months (pooled SMD: 0.53, 95% CI: 0.23–0.83, *p* < 0.05), as shown in Table 2. In the subgroup of frequency, the mind-body exercise showed a significant improvement in 6MWD compared to the control group for ≥5 times/week (pooled SMD: 0.72, 95% CI: 0.42–1.02, *p* < 0.05) and <5 times/week (pooled SMD: 0.38, 95% CI: 0.17–0.59, *p* < 0.05). In the subgroup of exercise duration, the mind-body exercise showed a significant improvement in 6MWD compared to the control group for ≥50 min (pooled SMD: 0.41, 95% CI: 0.16–0.65, *p* < 0.05) and <50 min (pooled SMD: 0.66, 95% CI: 0.39–0.93, *p* < 0.05).

Consistency and inconsistency tests were conducted on all *P*-values for indirect and direct comparisons among the studies, with all *P*-values greater than 0.05, indicating that the influence of consistency among the studies is acceptable. Details will be provided in Supplementary Table S7.

The results of the network meta-analysis indicate that the Liuzijue group [MD = 35.03, 95% CI = (6.55, 63.50)], Baduanjin group [MD = 30.84, 95% CI = (10.14, 51.54)], and the Tai Chi group [MD = 18.55, 95% CI = (1.65, 35.46)] significantly improved the 6MWD compared to the control group. Compared to Liuzijue [MD = 66.08, 95% CI = (15.54, 116.61)], Baduanjin [MD = 70.27, 95% CI = (24.02, 116.51)], Yijinjing [MD = 79.26, 95% CI = (8.85, 149.67)], Tai Chi [MD = 82.55, 95% CI = (37.53, 127.58)], Qigong [MD = 84.70, 95% CI = (35.38, 134.01)], Wuqinxi [MD = 110.46, 95% CI = (39.05, 181.87)], control [MD = 101.10, 95% CI = (59.78, 142.43)], and BWM [MD = 118.04, 95% CI = (41.37, 194.71)], Yoga demonstrated a statistically significant effect on the increase in 6MWD. In terms of increasing 6MWD, the probability rankings for different exercise interventions indicate that Yoga ranked highest in SUCRA (SUCRA: 99.7%, as shown in Figure 3C), followed by the Liuzijue, Baduanjin, Yijinjing, Tai Chi, Qigong, Wuqinxi, CON, and BWM (Table 3B; Figure 3C). As for Yoga, pooling evidence for the 6MWD showed an increase of 101.10 m (95% CI 59.78–142.43) and successed to reach the MCID of 26 m.

**Table 3B:** League table on 6MWD.

Yoga	Liuzijue	Baduanjin	Yijinjing	Tai Chi	Qigong	Wuqinxi	CON	BWM
**Yoga**	**−66.08** (**−116.61, −15.54)**	**−70.27** (**−116.51, −24.02)**	**−79.26** (**−149.67, −8.85)**	**−82.55** (**−127.58, −37.53)**	**−84.70** (**−134.01, −35.38)**	**−110.46** (**−181.87, −39.05)**	**−101.10** (**−142.43, −59.78)**	**−118.04** (**−194.71, −41.37)**
**66.08** (**15.54, 116.61)**	**Liuzijue**	−4.19 (−39.37, 30.99)	−13.18 (−76.91, 50.55)	−16.47 (−49.30, 16.35)	−18.62 (−57.88, 20.64)	−44.38 (−109.21, 20.45)	**−35.03** (**−63.50, −6.55)**	−51.96 (−122.21, 18.29)
**70.27** (**24.02, 116.51)**	4.19 (−30.99, 39.37)	**Baduanjin**	−8.99 (−69.64, 51.66)	−12.28 (−38.98, 14.41)	−14.43 (−48.45, 19.59)	−40.19 (−102.00, 21.62)	**−30.84** (**−51.54, −10.14)**	−47.77 (−115.22, 19.68)
**79.26** (**8.85, 149.67)**	13.18 (−50.55, 76.91)	8.99 (−51.66, 69.64)	**Yijinjing**	−3.29 (−62.76, 56.18)	−5.44 (−68.52, 57.64)	−31.20 (−112.70, 50.30)	−21.84 (−78.86, 35.17)	−38.78 (−124.75, 47.19)
**82.55** (**37.53, 127.58)**	16.47 (−16.35, 49.30)	12.28 (−14.41, 38.98)	3.29 (−56.18, 62.76)	**Tai Chi**	−2.15 (−34.03, 29.74)	−27.91 (−88.56, 32.74)	**−18.55** (**−35.46, −1.65)**	−35.49 (−101.91, 30.93)
**84.70** (**35.38, 134.01)**	18.62 (−20.64, 57.88)	14.43 (−19.59, 48.45)	5.44 (−57.64, 68.52)	2.15 (−29.74, 34.03)	**Qigong**	−25.76 (−89.95, 38.43)	−16.41 (−43.40, 10.59)	−33.34 (−103.14, 36.45)
**110.46** (**39.05, 181.87)**	44.38 (−20.45, 109.21)	40.19 (−21.62, 102.00)	31.20 (−50.30, 112.70)	27.91 (−32.74, 88.56)	25.76 (−38.43, 89.95)	**Wuqinxi**	9.36 (−48.89, 67.60)	−7.58 (−94.38, 79.21)
**101.10** (**59.78, 142.43)**	**35.03** (**6.55, 63.50)**	**30.84** (**10.14, 51.54)**	21.84 (−35.17, 78.86)	**18.55** (**1.65, 35.46)**	16.41 (−10.59, 43.40)	−9.36 (−67.60, 48.89)	**CON**	−16.94 (−81.50, 47.63)
**118.04** (**41.37, 194.71)**	51.96 (−18.29, 122.21)	47.77 (−19.68, 115.22)	38.78 (−47.19, 124.75)	35.49 (−30.93, 101.91)	33.34 (−36.45, 103.14)	7.58 (−79.21, 94.38)	16.94 (−47.63, 81.50)	**BWM**

Effect sizes where statistical differences exist are bolded.

#### 3.4.3 St. George's respiratory questionnaire (SGRQ)

**Figure 4:**
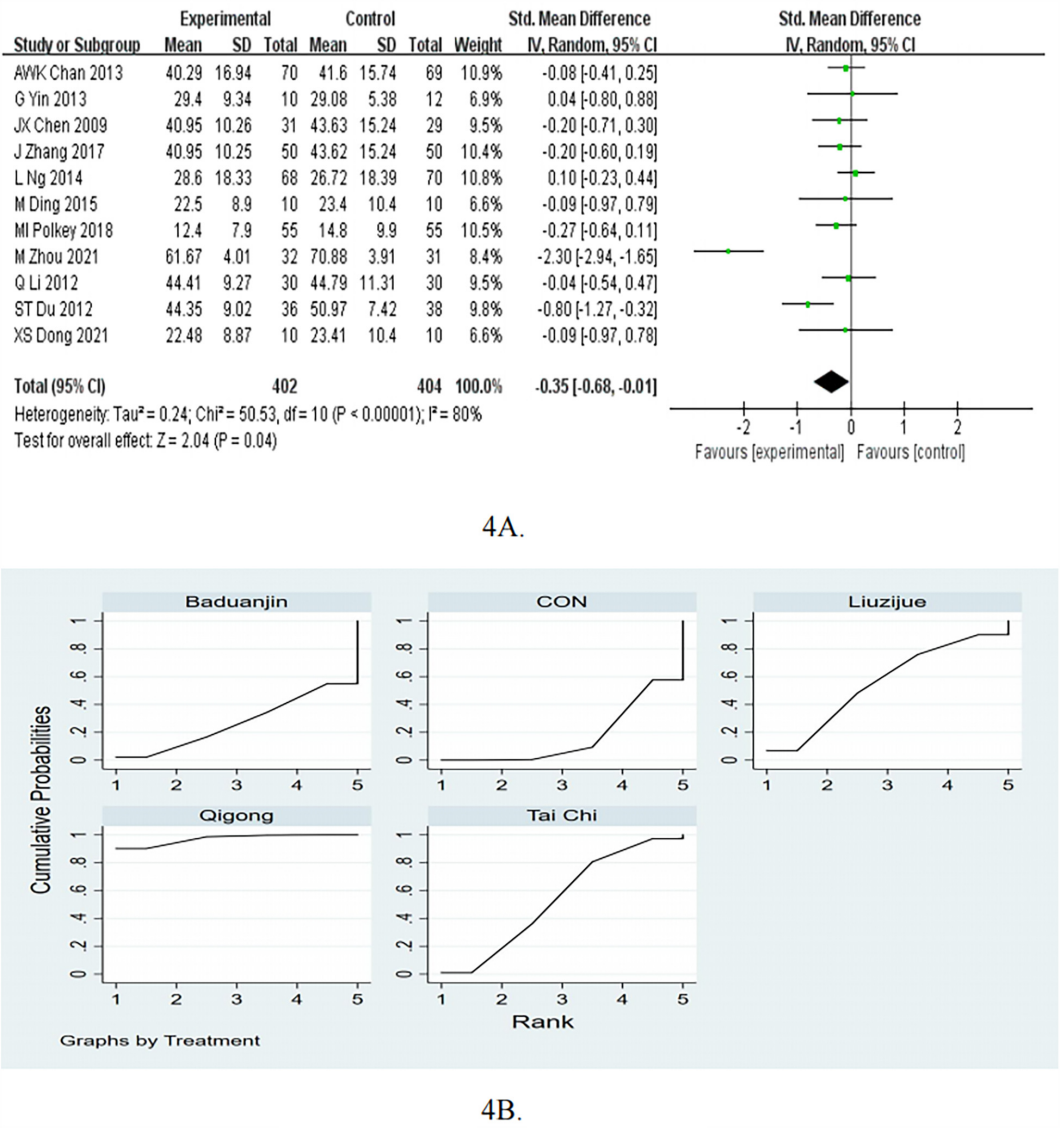
**(A)** Forest plots for the effects of mind-body exercise on SGRQ. **(B)** SUCRA plot for SGRQ.

The pooled results of the 11 studies showed that the SGRQ was significantly improved in the mind-body exercise group (SMD = −0.35, 95%CI −0.68 to −0.01, *p* < 0.05, *I*^2^ = 80%; Figure 4A).

In the subgroup of follow up, the mind-body exercise showed a significant improvement in SGRQ compared to the control group for ≤3months (pooled SMD: −0.32, 95% CI: −0.52 to −0.12, *p* < 0.05). As for >3 months, compared with the control intervention, mind-body exercise did not significantly reduce SGRQ (SMD = −0.43, 95%CI = −1.14 to 0.27, *P* = 0.23), as shown in Table 2. In the subgroup of frequency, the mind-body exercise did not significant reduce SGRQ score compared to the control group for ≥5 times/week (pooled SMD: −0.48, 95% CI: −1.06 to 0.09, *p* = 0.1) and <5 times/week (pooled SMD: −0.19, 95% CI: −0.53 to 0.15, *p* = 0.28). In the subgroup of exercise duration, the mind-body exercise did not significant reduce SGRQ score compared to the control group for ≥50 min (pooled SMD: −0.52, 95% CI: −1.06 to 0.02, *p* = 0.06) and ≥50 min (pooled SMD: −0.16, 95% CI: −0.42 to 0.10, *p* = 0.24).

Consistency and inconsistency tests were conducted on all *P*-values for indirect and direct comparisons among the studies, with most *P*-values greater than 0.05, indicating that the influence of consistency among the studies is acceptable. Details will be provided in Supplementary Table S8.

The results of the network meta-analysis indicate that Qigong was more effective than the Tai Chi [MD = −5.37, 95% CI = (−10.46, −0.28)], Baduanjin [MD = −7.52, 95% CI = (−15.00, −0.03)], and the control group [MD = −7.68, 95% CI = (−12.22, −3.15)] in improving SGRQ (Table 3C). In terms of increasing SGRQ, the probability rankings for different exercise interventions indicate that Qigong ranked first in SUCRA (SUCRA: 97.1%, as shown in Figure 4B). Compared to the control group, Qigong showed a decrease of −7.68 (95% CI −12.22 to −3.15) and reach the MCID.

**Table 3C:** League table on SGRQ.

Qigong	Liuzijue	Tai Chi	Baduanjin	CON
**Qigong**	5.01 (−1.71, 11.73)	**5.37** (**0.28, 10.46)**	**7.52** (**0.03, 15.00)**	**7.68** (**3.15, 12.22)**
−5.01 (−11.73, 1.71)	**Liuzijue**	0.36 (−5.34, 6.06)	2.51 (−5.23, 10.24)	2.67 (−2.29, 7.64)
**−5.37** (**−10.46, −0.28)**	−0.36 (−6.06, 5.34)	**Tai Chi**	2.15 (−4.42, 8.72)	2.32 (−0.48, 5.12)
**−7.52** (**−15.00, −0.03)**	−2.51 (−10.24, 5.23)	−2.15 (−8.72, 4.42)	**Baduanjin**	0.17 (−5.77, 6.10)
**−7.68** (**−12.22, −3.15)**	−2.67 (−7.64, 2.29)	−2.32 (−5.12, 0.48)	−0.17 (−6.10, 5.77)	**CON**

Effect sizes where statistical differences exist are bolded.

#### 3.4.4 Chronic obstructive pulmonary disease (COPD) assessment test (CAT)

**Figure 5:**
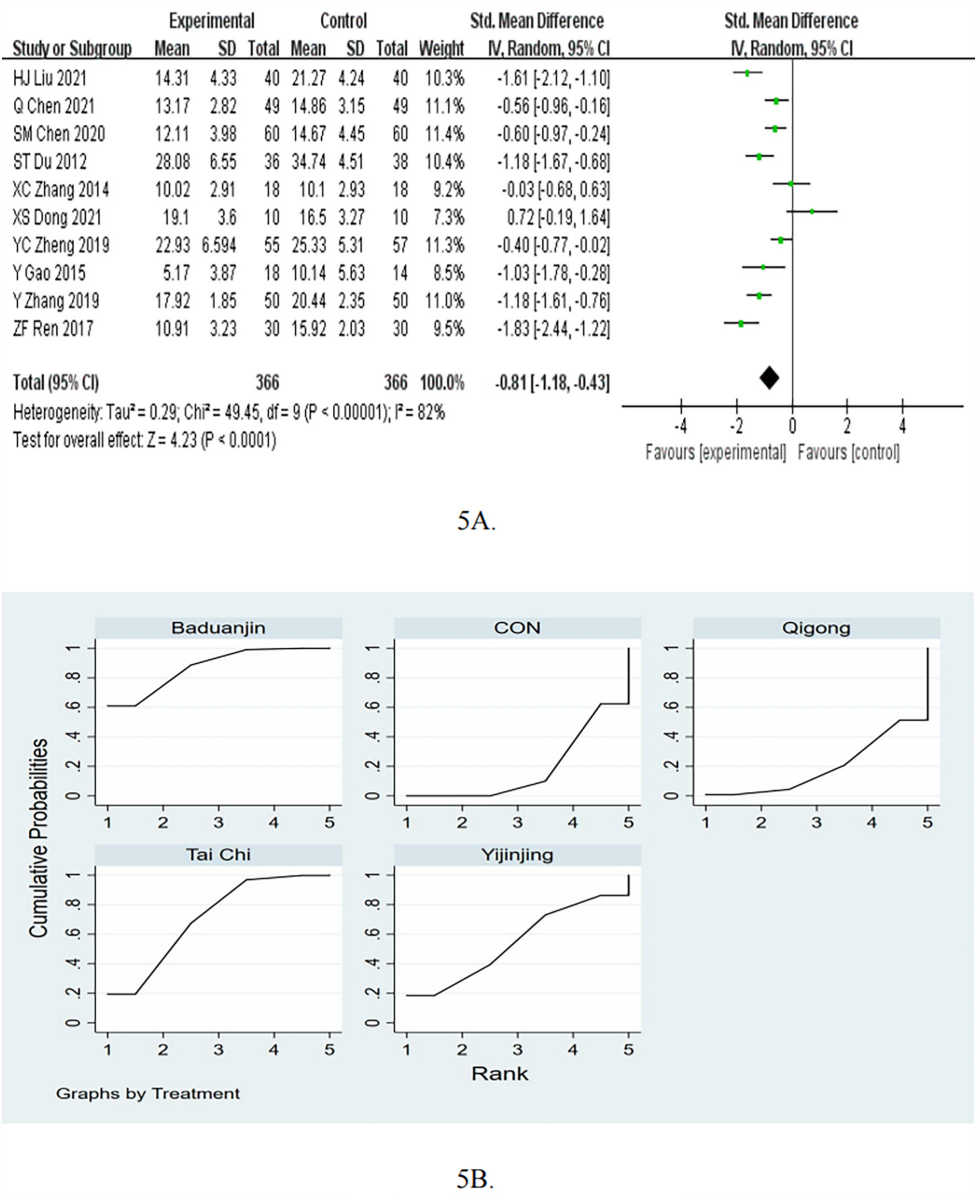
**(A)** Forest plots for the effects of mind-body exercise on CAT. **(B)** SUCRA plot for CAT.

The meta-analysis of the 10 RCTs showed that mind-body exercise was effective in reduce CAT (SMD = −0.81, 95%CI −1.18 to −0.43, *p* < 0.05, *I*^2^ = 82%; Figure 5A).

In the subgroup of follow up, the mind-body exercise showed a significant improvement in CAT compared to the control group for >3months (pooled SMD: −0.74, 95% CI: −1.14 to −0.34, *p* < 0.05). As for ≤3months, compared with the control intervention, mind-body exercise did not significantly reduce CAT (SMD = −0.88, 95%CI = −1.77 to 0.00, *P* = 0.05), as shown in Table 2. In the subgroup of frequency, the mind-body exercise showed a significant improvement in CAT compared to the control group for ≥5 times/week (pooled SMD: −0.77, 95% CI: −1.13 to −0.41, *p* < 0.05). As for <5 times/week, compared with the control intervention, mind-body exercise did not significantly reduce CAT (SMD = −0.82, 95%CI = −2.03 to 0.40, *P* = 0.19). In the subgroup of exercise duration, the mind-body exercise showed a significant improvement in CAT compared to the control group for ≥50 min (pooled SMD: −0.92, 95% CI: −1.48 to −0.36, *p* < 0.05) and <50 min (pooled SMD: −0.68, 95% CI: −1.25 to −0.11, *p* < 0.05).

Consistency and inconsistency tests were conducted on all *P*-values for indirect and direct comparisons among the studies, with most *P*-values greater than 0.05, indicating that the influence of consistency among the studies is acceptable. Details will be provided in Supplementary Table S9.

Figure 5B; Table 3D present the pooled MDs and 95%CIs for CAT among different interventions in the NMA. Compared to the control group, Baduanjin [MD = −4.78, 95% CI = (−7.90, −1.66)] and Tai Chi [MD = −3.49, 95% CI = (−6.08, −0.89)] demonstrated a statistically significant effect in reducing CAT. Additionally, Baduanjin [MD = −4.95, 95% CI = (−9.87, −0.03)] was more effective than Qigong. Based on the SUCRA rankings, Baduanjin had the highest probability of being the most effective therapy for reducing CAT, followed by the Tai Chi, Yijinjing, Qigong, and control group. As for Baduanjin, pooling evidence for the CAT showed a decrease of 4.78 and reach the MCID of 3 points.

**Table 3D:** League table on CAT.

Baduanjin	Tai Chi	Yijinjing	Qigong	CON
**Baduanjin**	1.29 (−2.77, 5.35)	2.38 (−3.83, 8.59)	**4.95** (**0.03, 9.87)**	**4.78** (**1.66, 7.90)**
−1.29 (−5.35, 2.77)	**Tai Chi**	1.09 (−4.88, 7.05)	3.66 (−0.95, 8.26)	**3.49** (**0.89, 6.08)**
−2.38 (−8.59, 3.83)	−1.09 (−7.05, 4.88)	**Yijinjing**	2.57 (−4.00, 9.15)	2.40 (−2.97, 7.77)
**−4.95** (**−9.87, −0.03)**	−3.66 (−8.26, 0.95)	−2.57 (−9.15, 4.00)	**Qigong**	−0.17 (−3.97, 3.62)
**−4.78** (**−7.90, −1.66)**	**−3.49** (**−6.08, −0.89)**	−2.40 (−7.77, 2.97)	0.17 (−3.62, 3.97)	**CON**

Effect sizes where statistical differences exist are bolded.

#### 3.4.5 N-terminal pro-B-type natriuretic peptide (NT-proBNP)

**Figure 6:**
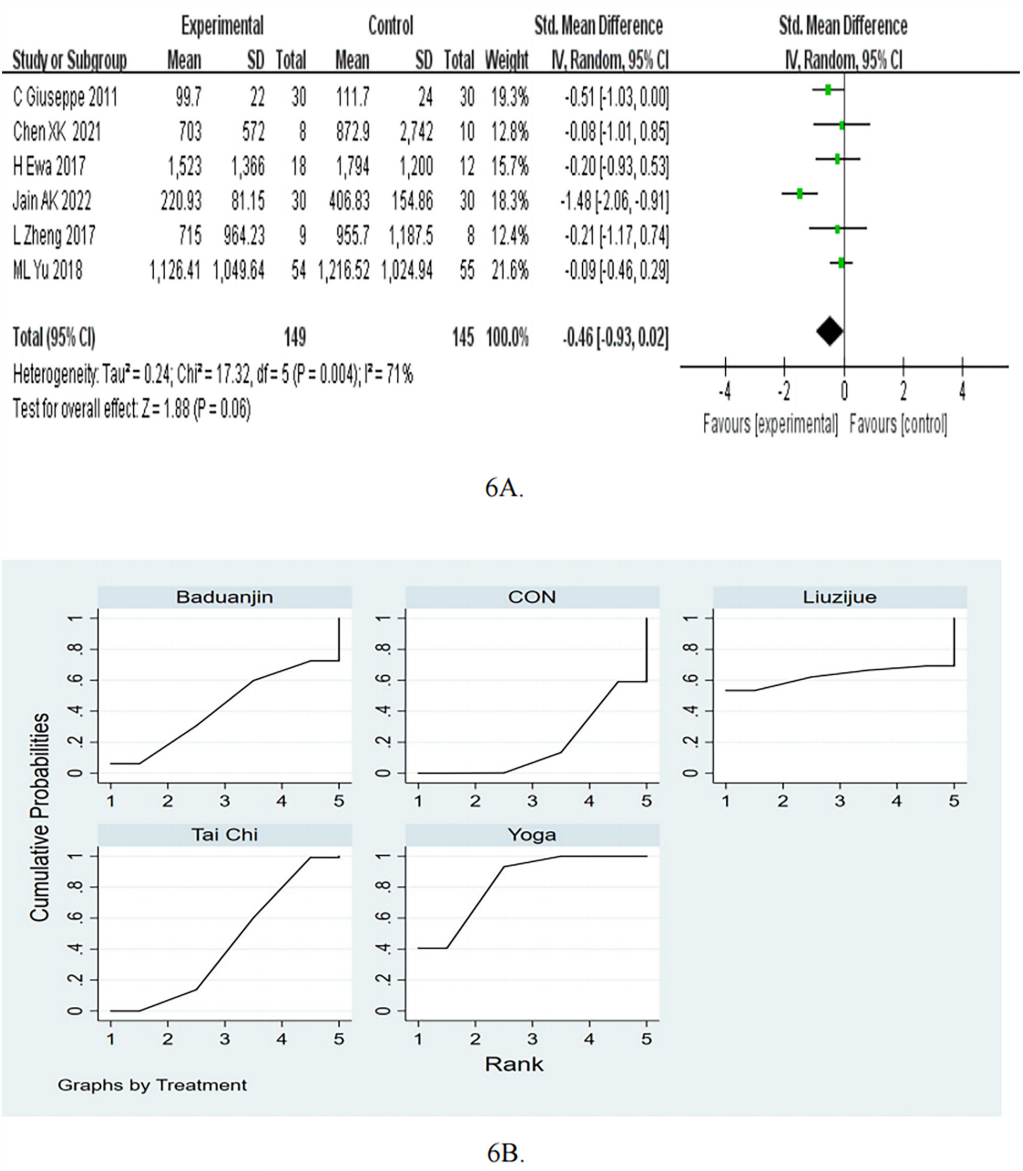
**(A)** Forest plots for the effects of mind-body exercise on NT-proBNP. **(B)** SUCRA plot for NT-proBNP.

Compared with the control intervention, mind-body exercise did not significantly reduce NT-proBNP (RCTs: *n* = 6; SMD = −0.46, 95%CI = −0.93 to 0.02, *P* = 0.06, I^2^ = 71%, Figure 6A).

In the subgroup of frequency, the mind-body exercise did not significant reduce NT-proBNP compared to the control group for ≥5 times/week (pooled SMD: −0.65, 95% CI: −1.64 to 0.34, *p* = 0.2) and <5 times/week (pooled SMD: −0.23, 95% CI: −0.51 to 0.05, *p* = 0.11), as shown in Table 2. In the subgroup of exercise duration, the mind-body exercise showed a significant improvement in NT-proBNP compared to the control group for ≥50 min (pooled SMD: −0.75, 95% CI: −1.49 to −0.01, *p* < 0.05). As for <50 min, compared with the control intervention, mind-body exercise did not significantly reduce NT-proBNP (SMD = −0.10, 95%CI = −0.43 to 0.23, *P* = 0.55).

Consistency and inconsistency tests were conducted on all *P*-values for indirect and direct comparisons between the studies, with all *P*-values greater than 0.05, indicating that the influence of consistency among the studies is acceptable. Details will be listed in Supplementary Table S10.

The results of the network meta-analysis indicate that Yoga [MD = −186.29, 95% CI = (−248.71, −123.87)] and Tai Chi [MD = −12.00, 95% CI = (−23.65, −0.35)] were more effective than control group in reducing the NT-proBNP. Moreover, Yoga [MD = −174.29, 95% CI = (−237.78, −110.79)] was more effective than Tai Chi. Based on the SUCRA rankings, Yoga had the highest probability of being the most effective therapy for reducing NT-proBNP, followed by Liuzijue, Tai Chi, Baduanjin, and control (Table 3E; Figure 6B). As for NT-proBNP, compared to the control group, Yoga showed a decrease of −186.29 (95% CI −248.71 to −123.87, *p* < 0.05).

**Table 3E:** League table on NT-proBNP.

Yoga	Liuzijue	Tai Chi	Baduanjin	CON
**Yoga**	−54.41 (−1,092.62, 983.79)	**174.29** (**110.79, 237.78)**	148.80 (−99.42, 397.03)	**186.29** (**123.87, 248.71)**
54.41 (−983.79, 1, 092.62)	**Liuzijue**	228.70 (−807.69, 1, 265.09)	203.22 (−860.59, 1, 267.03)	240.70 (−795.63, 1, 277.03)
**−174.29** (**−237.78, −110.79)**	−228.70 (−1, 265.09, 807.69)	**Tai Chi**	−25.48 (−266.02, 215.05)	**12.00** (**0.35, 23.65)**
−148.80 (−397.03, 99.42)	−203.22 (−1, 267.03, 860.59)	25.48 (−215.05, 266.02)	**Baduanjin**	37.48 (−202.77, 277.73)
**−186.29** (**−248.71, −123.87)**	**−240.70** (**−1, 277.03, 795.63)**	−12.00 (−23.65, −0.35)	−37.48 (−277.73, 202.77)	**CON**

League table for outcomes: A: league table on MLHFQ; B: league table on 6MWD; C: league table on SGRQ; D: league table on CAT; E: league table on NT-proBNP. BWM: Buddhist walking meditation, MLHFQ: Minnesota Living with Heart Failure Questionnaire, 6MWD: 6-minute walk distance, SGRQ: St. George's Respiratory Questionnaire; CAT: Chronic Obstructive Pulmonary Disease (COPD) Assessment Test, NT-proBNP: N-terminal pro-B-type natriuretic peptide.

Effect sizes where statistical differences exist are bolded.

### 3.5 Publication bias test

**Figure 7:**
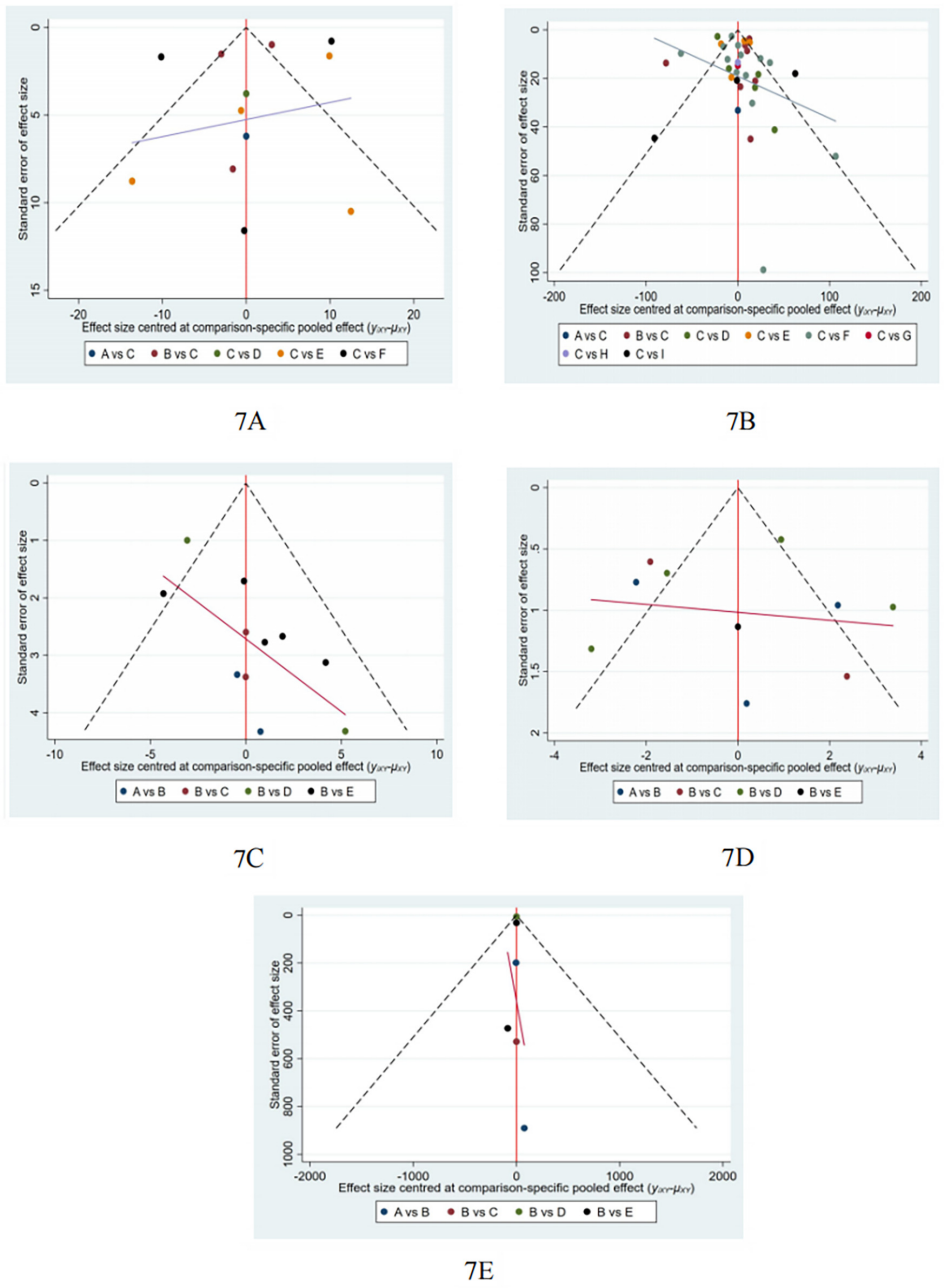
Funnel plot on publication bias. **(A)**: MLHFQ; **(B)**: 6MWD; **(C)**: SGRQ; **(D)**, CAT; **(E)**: NT-proBNP.

We constructed separate funnel plots for all outcome measures to test for potential publication bias. Visual inspection of the funnel plots revealed no significant publication bias. Details are shown in Figure 7. The findings of Egger's and Begg's tests to identify study bias (Table 4). From the results, it could be considered that there was no publication bias.

**Table 4:** The findings of Egger's and Begg's tests to identify study bias.

Outcomes	Study bias	
	Egger's test	Begg's test
MLHFQ	0.516	0.583
6MWD	0.732	0.764
SGRQ	0.355	0.392
CAT	0.962	0.929
NT-ProBNP	0.852	0.851

*p* > 0.05, no publication bias. MLHFQ, Minnesota living with heart failure questionnaire; 6MWD, 6-minute walk distance; SGRQ, St. George's respiratory questionnaire; CAT, chronic obstructive pulmonary disease (COPD) assessment test; NT-proBNP, N-terminal pro-B-type natriuretic peptide.

## 4 Discussion

In this study, we compared the effects of different exercise interventions on the rehabilitation of patients with chronic cardiopulmonary dyspnoea. A total of 44 studies were included, encompassing eight different exercise protocols and involving 2,957 patients diagnosed with chronic cardiopulmonary dyspnoea, which constitutes a substantial sample size. Firstly, our study suggests that Yoga may be the most effective exercise intervention to reduce MLHFQ scores (SUCRA: 71.8%). Secondly, Yoga training demonstrated better results in increasing the 6MWD (SUCRA: 99.7%). Thirdly, Qigong was also identified as the most effective exercise intervention for decreasing SGRQ (SUCRA: 97.1%). Fourthly, compared to other treatment methods, Baduanjin may be the most suitable intervention for lowering CAT (SUCRA: 87.1%). Fifthly, Yoga might be more advantageous for decreasing the NT-proBNP (SUCRA: 83.4%).

The findings of this NMA are largely consistent with the findings of other recently published systematic reviews for chronic cardiopulmonary dyspnoea (90–92). Zhang et al. evaluate whether traditional mind-body therapy can be used as a complementary or alternative therapy for chronic cardiopulmonary dyspnoea (90). This systematic review enrolled only 34 studies involving 2,456 patients (90). This meta-analysis revealed that mind-body exercise significant improvements in the 6MWD for COPD and HF patients (90). Besides, Luo et al. revealed that mind-body exercises could significant improvements in the 6MWD for COPD patients (92). However, in this current NMA, study number and sample size were significantly increased (44 studies enrolling 2,957 patients). Moreover, many more types of outcomes have been evaluated, such as MLHFQ score, SGRQ, CAT, and NT-proBNP. Sujie, M. et al. revealed that mind-body exercises can reduced COPD patients' SGRQ score and CAT score (91). This is similar to our study. In our study, we revealed that Qigong was also identified as the most effective exercise intervention for decreasing SGRQ. When compared with other treatments, Baduanjin is likely the most appropriate intervention for decreasing CAT.

Our study indicates that Yoga is the most effective exercise intervention for reducing the MLHFQ scores. Yoga is a mind-body intervention that purposefully influences mental and physical health through concepts related to autonomic function (93). These findings are consistent with previous research. Previous studies have shown that MLHFQ scores are associated with depressive symptoms, and lower HRQoL is linked to a higher incidence of depression (94). The significant improvement of Yoga on MLHFQ score of patients with heart failure stems from its multi-target regulation of the autonomic nervous system and the immune system. Slow abdominal breathing, which is unique to Yoga, can enhance the tension of the cardiac vagus nerve (95). Acetylcholine released from vagus nerve endings can specifically bind to alpha7 acetylcholine receptor (α7Achr) (96, 97) inhibits the phosphorylation of the NF-κB signaling pathway, thus reducing the release of pro-inflammatory factors TNF-α and IL−6 (98). Clinical research shows that the decrease of inflammatory factors is directly related to the improvement of patients' fatigue and sitting breathing (98). Previous studies have shown that the hippocampal *α*7nAchr/NF-κB signal pathway is related to the antidepressant effect, and the depression score is also one of the MLHFQ scale (97). Taken together, these results suggest that Yoga may serve as a novel therapeutic approach for improving physical and mental function in elderly individuals (99).

Yoga training demonstrated better results in improving the 6MWD. These findings are consistent with previous research. Meta-analyses have shown a significant improvement in the 6MWD associated with Yoga (100). Results from Krishna et al. indicated that the Yoga group had a significant increase in 6MWD compared to the control group (88). Similarly, Jayadevappa et al. found significant improvements in 6MWD in the Yoga group at 6 months post-treatment compared to the health education group (101). The 6MWD has been shown to independently predict mortality, making it an important surrogate marker in the absence of hard clinical endpoints (102, 103). Numerous researchers have investigated how Yoga affects hypertension (104). A thorough meta-exploration of 44 randomized controlled trials found that nine studies showed Yoga to be more effective than traditional treatments in improving blood pressure (104). Measurements of diastolic and systolic blood pressure averaged reductions of 6 mmHg and 4 mmHg, respectively (105). Previous studies found that a Yoga therapy program, which included asanas and breath control, significantly reduced both systolic blood pressure (SBP) and diastolic blood pressure (DBP) (106–109). Although not a hard clinical endpoint, it is known that reductions in blood pressure translate to fewer cardiovascular events in other cardiovascular populations (110), and improvements in 6MWD are associated with lower cardiovascular mortality (111, 112). A community study focused on elderly populations observed a significant relationship between persistently elevated systolic blood pressure and the incidence of heart failure (113). During heart failure, sympathetic nervous activity is significantly increased and persists for a longer duration (113). Concurrently, the withdrawal of the vagus nerve is also an important aspect of heart failure (108). Elevated blood pressure is a strong predictor of outcomes in congestive heart failure (114–116). In addition to hemodynamic disturbances, increased neurohormonal activation through the sympathetic nervous system and the renin-angiotensin system is also associated with the progression of heart failure (117, 118). Previous studies have shown that adding Yoga therapy to standard medical treatment leads to significant reductions in blood pressure, resulting in a shift towards vagal dominance (108). Yoga is thought to improve baroreceptor sensitivity while reducing sympathetic activity, serving as a potential strategy for lowering blood pressure, which has significant implications for heart failure (104, 119). In addition to physical activity, Yoga also incorporates breathing exercises, relaxation techniques, and meditation (100). It is believed that breathing, meditation, and relaxation techniques may enhance parasympathetic activity, reduce sympathetic activity, and improve baroreflex sensitivity (119). By increasing baroreflex sensitivity and heart rate variability, a lower breathing frequency can facilitate vagal activation and diminish the effects of the sympathetic branch of the autonomic nervous system (100). As vagal impairment increases, both blood pressure and heart rate may decline (100). In addition, Yoga up-regulates the activity of endothelial nitric oxide synthase (eNOS) (120), reduces the peripheral vascular resistance through endothelium-dependent vasodilatation, and further optimizes the oxygen transport-utilization matching (121–123).

We implemented CAT and SGRQ scores, which are commonly used in the clinic to assess the QoL in patients with COPD. Baduanjin is also the most effective exercise intervention for decreasing CAT. Baduanjin, as a traditional aerobics exercise, is listed as the 97th sports item officially launched by the State General Administration of Sport in 2003 and has been widely promoted in China (124). Previous studies have confirmed that Baduanjin has a strong clinical effect on lung rehabilitation in COPD patients (125). Thus, Baduanjin has become an exercise prescription for COPD (126). These findings are consistent with previous research. Shuai et al. revealed that Baduanjin exercise may have the potential to enhance mental status, exercise ability, health status, quality of life, and lung function of COPD patients (127). The CAT scale, developed based on the St. George's Respiratory Questionnaire, has been shown to reliably assess the health status of COPD patients and has validity in measuring the effects of pulmonary rehabilitation and the impact of exercise interventions (128–130). Improvements in exercise capacity, lung function, and quality of life of patients with COPD may be closely related to the exercise patterns of the Baduanjin. The upper limb stretching and trunk extension movements of Baduanjin, as well as the combination of thoracic and abdominal respiration, effectively exercise the pectoral muscles, diaphragm, and abdominal muscles, thereby enhancing respiratory muscle contraction and improving lung capacity (131, 132). Baduanjin also combines mental focus and physical relaxation to reduce patient fatigue and increase psychological pleasure, thus enhancing exercise compliance (126, 133). In addition, the upper and lower extremity synergistic movements of Baduanjin can strengthen the assistive respiratory muscles, improve respiratory efficiency by increasing gas exchange, delay the deterioration of lung function, and reduce the levels of IL−8 and C-reactive protein in sputum to alleviate the inflammatory response (125, 134–136). Long-term training can improve the patient's respiratory muscle strength and endurance, thus improving respiratory function and alleviating the symptoms of dyspnea (136).

This NMA showed that Yoga showed better results in improving NT-proBNP. These findings are consistent with previous studies. The meta-analysis showed that Yoga improves NT-proBNP (137). The mechanism is closely related to the regulation of neuroendocrine and cardiovascular function. NT-proBNP, an inactive precursor of brain natriuretic peptide (BNP), is secreted by the ventricles in response to volume loading or pressure overload, and its elevated concentration is an important marker for the diagnosis of heart failure and assessment of the disease (138, 139). Studies have shown that Yoga, through a combination of asana training, breath control, and meditation, enhances parasympathetic activity and decreases sympathetic activity, which reduces ventricular filling pressures and myocardial wall tension-all key factors in stimulating NT-proBNP release (137, 140, 141). In addition, Yoga stretches can increase the efficiency of oxygen uptake by peripheral tissues, improve muscle strength, endurance and flexibility, and improve oxygen uptake, thereby reducing myocardial metabolic load and cardiovascular risk (137, 142, 143). Long-term practice can also reduce the secretion of stress hormones such as catecholamines, cortisol and aldosterone by regulating the hypothalamic-pituitary-adrenal axis, further alleviating the cardiac load and inflammatory response, and further inhibiting the release of NT-proBNP (46, 143). The synergistic effect of the above multiple pathways ultimately reduces NT-proBNP levels, suggesting that Yoga may provide a non-pharmacological intervention strategy for cardiovascular disease management by improving cardiac function and neuroendocrine homeostasis. Activation of the vagus nerve suppresses the renin-angiotensin-aldosterone system (RAAS), leading to decreased plasma renin activity and angiotensin II levels, which in turn reduces ventricular preload (144). Concurrently, Yoga enhances endothelial nitric oxide synthase (eNOS) activity, promoting endothelium-dependent vasodilation (120, 123). This mechanism lowers peripheral vascular resistance, reduces mean arterial pressure and vascular resistance index, and ultimately decreases ventricular wall stress (120, 123). Both of these pathways are closely associated with changes in NT-proBNP levels. However, future studies with larger samples are needed to demonstrate the above results.

Our research shows that Yoga may be the most effective mind-body exercise intervention method to improve MLHFQ, 6MWD and NT-proBNP. At present, there is no study on the economic benefits of Yoga in treating chronic cardiopulmonary dyspnoea, and it is impossible to make a horizontal comparison through QALY and other indexes. Philipson et al. demonstrated that Yoga interventions for young girls with functional abdominal pain disorders generate small quality-adjusted life years (QALYs) gains and monetary savings compared to standard healthcare, suggesting potential cost-effectiveness (145). Tew et al. found that Yoga was associated with an additional cost of £80.85 per participant and yielded an incremental 0.0178 QALYs per participant on average compared to usual care, indicating cost-effectiveness at conventional willingness-to-pay thresholds (146). Hartfiel et al. reported that, from a healthcare system perspective, Yoga interventions for musculoskeletal conditions incurred higher total costs than usual care due to implementation expenses (£54.52 more per participant), though healthcare resource utilization costs were £20.86 lower per person in the yoga group (147). Previous studies further revealed that Yoga was cost-effective from an organizational perspective and dominant from a societal perspective, delivering both health benefits and cost savings (148). Collectively, these studies suggest that Yoga interventions are associated with reduced healthcare resource utilization costs. Future research can carry out Yoga to analyze the economic benefits of patients with chronic cardiopulmonary dyspnoea to guide clinical application.

In the process of analysis, we conducted subgroup analysis according to follow-up, frequency and exercise duration. Our subgroup analyses stratified by follow-up duration, exercise frequency, and exercise duration consistently demonstrated that mind-body exercise produced statistically significant improvements in both 6MWD and MLHFQ scores compared to control interventions. These findings suggest the therapeutic benefits of mind-body exercise on 6MWD and MLHFQ are robust across varying implementation parameters. While the meta-analysis demonstrated significant improvements in SGRQ scores with mind-body exercise compared to controls, subsequent subgroup analyses stratified by intervention frequency (≥5 times/week vs. <5 times/week) and exercise duration (≥50 min vs. <50 min) revealed non-significant between-group differences. The observed attenuation of the therapeutic effects in the implementation parameters of the scheme indicates that the preliminary findings may need careful interpretation, which may reflect the sample size limitations in subgroup comparisons or the different reactivity based on exercise dose parameters. Meta-analysis showed that compared with the control group, the CAT score in the mind-body exercise group was significantly improved. Subsequently, the subgroup analyses stratified by follow-up duration revealed differential effects: in the >3 months cohort, interventions showed clinically meaningful CAT score reduction, whereas the ≤3 months subgroup exhibited non-significant changes. This subgroup analysis indicates that the possible cumulative treatment effects require longer intervention durations to show measurable clinical impacts.

This systematic review and NMA has several advantages, including being the first study to evaluate the effects of mind-body exercises on patients with chronic cardiopulmonary dyspnoea. The strength of this study lies in its methodology, which adheres to PRISMA guidelines and employs a robust search strategy designed to capture all types of adaptive mind-body activities for patients with chronic cardiopulmonary dyspnoea. Secondly, the studies included in this review were conducted across different continents, including Asia, the Americas, and Europe, highlighting that mind-body exercises are a global practice with the potential for widespread dissemination and impact. One of the strengths of this review is that it is based on a comprehensive search of several databases, focusing exclusively on randomized controlled trials.

However, when interpreting our findings, it is important to consider some limitations of the studies. Firstly, over half the studies had moderate/high risk of bias. However, this is unavoidable since participants in the mind-body exercise group were aware of the treatment involved during the intervention, and using unblinded coaches during the intervention would be unethical for enhancing training efficacy and patient safety. Future research should address blinding limitations by using sham exercises or objective outcome assessors for the control group. Secondly, the significant improvements in mind-body exercises require careful interpretation due to the heterogeneity of study designs and the short duration of interventions. Lack of standardization across studies can lead to differences in results. In addition, there is heterogeneity in study design and interventions, which makes analysis tricky and potentially error-prone. Compared with large-scale trials, small-scale trials can exaggerate the curative effects. Follow-up studies should standardize the duration of exercise and intensity of treatment for mind-body exercise. Thirdly, exercise outcomes are influenced by various factors such as the duration of each intervention session, intervention frequency, intervention period, patient physical condition, and severity of illness. Due to the limitations of our methodological approach, we could only focus on the overall effects of mind-body exercises, without considering the impact of other exercise parameters such as frequency, duration, and intensity. This study included 44 RCTs to examine the effects of different mind-body exercises on patients with chronic cardiopulmonary dyspnoea, but these mind-body exercises had different styles, intensities, and instructor-led variations, which would have had an impact on outcome metrics. In addition, the placebo effect from positive expectations during the study could have biased the results. When conducting multiple comparisons, we were unable to control for other variables, such as gender and age differences in exercise types and experimental settings. Additionally, the included studies had difficulty controlling for all external variables as rigorously as laboratory studies: On one hand, the varying characteristics of mind-body practices, such as the level of Tai Chi postures, skill proficiency, and the seamless experience brought by the mind-body integration, can influence the effectiveness of the exercise interventions. On the other hand, variations in the duration of chronic cardiopulmonary dyspnoea and health status contribute to the variability in outcomes related to improvements in MLHFQ, 6MWD, SGRQ, CAT, and NT-proBNP among chronic cardiopulmonary dyspnoea patients.

## 5 Conclusion

This study aimed to conduct a network meta-analysis to compare the effectiveness of different mind-body exercise interventions on the rehabilitation of patients with chronic cardiopulmonary dyspnoea. Our findings indicate that Yoga might be the most effective exercise intervention for reducing the MLHFQ scores, increasing the 6MWD, and decreasing the NT-proBNP. Qigong is also identified as the most effective exercise intervention for decreasing SGRQ. Compared to other treatment methods, Baduanjin may be the most suitable intervention for lowering CAT. Although there are variations in the effects of the interventions, the rankings of their effectiveness need to be interpreted cautiously due to the quality limitations of the original studies. Therefore, our study recommends that patients with chronic cardiopulmonary dyspnoea select appropriate mind-body exercises based on their individual conditions and adhere to them under the guidance of healthcare professionals. Future research should conduct more high-quality randomized controlled trials to investigate the impact of various forms of mind-body exercise on the effective prevention and treatment of chronic cardiopulmonary dyspnoea. Follow-up studies should standardize the duration of exercise and intensity of treatment for mind-body exercise.

## Data Availability

The original contributions presented in the study are included in the article/Supplementary Material, further inquiries can be directed to the corresponding author.
