# Supplementary material for: Effects of mind-body exercise in chronic cardiopulmonary dyspnoea patients—a network meta-analysis of randomized controlled trials
Source: Front Cardiovasc Med. 2025 Jun 4;12:1546996. doi: 10.3389/fcvm.2025.1546996 (PMC12174109; doi:10.3389/fcvm.2025.1546996)
Supplement: Supplementary file 1 [file Table1.docx]

**Table S1. Search strategy**

Search date: November 21, 2024

Databases searched: PubMed, EMBASE, Web of Science, Cochrane Library, and China National Knowledge Infrastructure.

**Search Strategy in PUBMED**

| Search | PUBMED | Results |
| --- | --- | --- |
| #1 | (((((((((((((((((((Pulmonary Disease, Chronic Obstructive[MeSH Terms]) OR (Pulmonary Disease, Chronic Obstructive[Title/Abstract])) OR (Chronic Obstructive Pulmonary Diseases[Title/Abstract])) OR (COPD[Title/Abstract])) OR (Chronic Obstructive Lung Disease[Title/Abstract])) OR (Chronic Obstructive Pulmonary Disease[Title/Abstract])) OR (COAD[Title/Abstract])) OR (Chronic Obstructive Airway Disease[Title/Abstract])) OR (Airflow Obstruction, Chronic[Title/Abstract])) OR (Airflow Obstructions, Chronic[Title/Abstract])) OR (Chronic Airflow Obstructions[Title/Abstract])) OR (Chronic Airflow Obstruction[Title/Abstract])) OR (chronic lung resistance[Title/Abstract])) OR (chronic pulmonary obstruction[Title/Abstract])) OR ((((((((((((((pulmonary fibrosis[MeSH Terms]) OR (pulmonary fibrosis[Title/Abstract])) OR (interstitial lung disease[Title/Abstract])) OR (lung fibrosis[Title/Abstract])) OR (idiopathic pulmonary fibrosis[Title/Abstract])) OR (pulmonary interstitial fibrosis[Title/Abstract])) OR (Fibroses, Pulmonary[Title/Abstract])) OR (Fibrosis, Pulmonary[Title/Abstract])) OR (Pulmonary Fibroses[Title/Abstract])) ) OR (Alveolitis, Fibrosing[Title/Abstract])) OR (Alveolitides, Fibrosing[Title/Abstract])) OR (Fibrosing Alveolitides[Title/Abstract])) OR (Fibrosing Alveolitis[Title/Abstract])) OR (Idiopathic Diffuse Interstitial Pulmonary Fibrosis[Title/Abstract]))) OR (((((pulmonary artery hypertension[MeSH Terms]) OR (pulmonary artery hypertension[Title/Abstract])) OR (pulmonary hypertension[Title/Abstract])) OR (Hypertension, Pulmonary[Title/Abstract])) OR (Pulmonary Artery[Title/Abstract]))) OR ((((((((((pulmonary embolism[MeSH Terms]) OR (pulmonary embolism[Title/Abstract])) OR (pulmonary thromboembolism[Title/Abstract])) OR (lung embolism[Title/Abstract])) OR (Embolism, Pulmonary[Title/Abstract])) OR (Embolisms, Pulmonary[Title/Abstract])) OR (Pulmonary Embolisms[Title/Abstract])) OR (Pulmonary Thromboembolisms[Title/Abstract])) OR (Thromboembolisms, Pulmonary[Title/Abstract])) OR (Thromboembolism, Pulmonary[Title/Abstract]))) OR ((((((((((((Heart Valve Diseases[MeSH Terms]) OR (Heart Valve Diseases[Title/Abstract])) OR (Heart Valve Disease[Title/Abstract])) OR (Valve Disease, Heart[Title/Abstract])) OR (Valvular Heart Diseases[Title/Abstract])) OR (Heart Disease, Valvular[Title/Abstract])) OR (Valvular Heart Disease[Title/Abstract])) OR (Heart Valvular Disease[Title/Abstract])) OR (Disease, Heart Valvular[Title/Abstract])) OR (Heart Valvular Diseases[Title/Abstract])) OR (Valvular Disease, Heart[Title/Abstract])) OR (cardiac valve disease[Title/Abstract]))) OR (((((Cardiomyopathies[MeSH Terms]) OR (Cardiomyopathies[Title/Abstract])) OR (Cardiomyopathy[Title/Abstract])) OR (myocardial diseases[Title/Abstract])) OR (Myocardiopathies[Title/Abstract]))) OR ((((((((((((((((Heart Failure[MeSH Terms]) OR (Cardiac Failure[Title/Abstract])) OR (Heart Decompensation[Title/Abstract])) OR (Decompensation, Heart[Title/Abstract])) OR (Heart Failure, Right-Sided[Title/Abstract])) OR (Heart Failure, Right Sided[Title/Abstract])) OR (Right-Sided Heart Failure[Title/Abstract])) OR (Right Sided Heart Failure[Title/Abstract])) OR (Myocardial Failure[Title/Abstract])) OR (Congestive Heart Failure[Title/Abstract])) OR (Heart Failure, Congestive[Title/Abstract])) OR (Heart Failure, Left-Sided[Title/Abstract])) OR (Heart Failure, Left Sided[Title/Abstract])) OR (Left-Sided Heart Failure[Title/Abstract])) OR (Left Sided Heart Failure[Title/Abstract])) OR (Heart Failure[Title/Abstract])) | 824,177 |
| #2 | (((Yoga[MeSH Terms]) OR (Yoga[Title/Abstract])) OR ((((((((((((Tai Ji[MeSH Terms]) OR (Tai Ji[Title/Abstract])) OR (Tai-ji[Title/Abstract])) OR (Tai Chi[Title/Abstract])) OR (Chi, Tai[Title/Abstract])) OR (Tai Ji Quan[Title/Abstract])) OR (Ji Quan, Tai[Title/Abstract])) OR (Quan, Tai Ji[Title/Abstract])) OR (Taiji[Title/Abstract])) OR (Taijiquan[Title/Abstract])) OR (T'ai Chi[Title/Abstract])) OR (Tai Chi Chuan[Title/Abstract]))) OR ((((((((((Qigong[MeSH Terms]) OR (Qigong[Title/Abstract])) OR (Qi Gong[Title/Abstract])) OR (Ch'i Kung[Title/Abstract])) OR (mind-body exercise[Title/Abstract])) OR (baduanjin[Title/Abstract])) OR (Liuzijue[Title/Abstract])) OR (Yijinjing[Title/Abstract])) OR (Wuqinxi[Title/Abstract])) OR (Buddhist walking meditation[Title/Abstract])) | 11,818 |
| #3 | #1 AND #2 | 324 |
