# Supplementary material for: Effects of mind-body exercise in chronic cardiopulmonary dyspnoea patients—a network meta-analysis of randomized controlled trials
Source: Front Cardiovasc Med. 2025 Jun 4;12:1546996. doi: 10.3389/fcvm.2025.1546996 (PMC12174109; doi:10.3389/fcvm.2025.1546996)
Supplement: Supplementary file 2 [file Table2.docx]

**Table S2 Minnesota Living with Heart Failure Questionnaire**

| During the past month, has your heart problem prevented you from living as you wanted? Why? | | | | | | |
| --- | --- | --- | --- | --- | --- | --- |
| 1 | It caused swelling in your ankles and legs | | | | | |
| 2 | It made you sit or lie down to rest during the day | | | | | |
| 3 | It made walking and climbing stairs difficult | | | | | |
| 4 | It made your work around the house difficult | | | | | |
| 5 | It made going places away from home difficult | | | | | |
| 6 | It made it difficult to sleep well at night | | | | | |
| 7 | It made your relationships or activities with family and friends difficult | | | | | |
| 8 | It made your work to earn a living difficult | | | | | |
| 9 | It made your recreational pastimes, sports or entertainment/hobbies difficult | | | | | |
| 10 | It made your sexual activities difficult | | | | | |
| 11 | It made you eat less of the foods you like | | | | | |
| 12 | It caused shortness of breath | | | | | |
| 13 | It made you tired,fatigued,or low on energy | | | | | |
| 14 | It made you stay in a hospital | | | | | |
| 15 | It caused you to spend money for medical care | | | | | |
| 16 | It caused side effects from the medications | | | | | |
| 17 | It made you feel you are a burden to your family or friends | | | | | |
| 18 | It made you feel a loss of self-control in your life | | | | | |
| 19 | It made you worry | | | | | |
| 20 | It made it difficult for you to concentrate or remember things | | | | | |
| 21 | It made you feel depressed | | | | | |
| Score | No | Very Little |  |  |  | Very Much |
|  | 0 | 1 | 2 | 3 | 4 | 5 |
