# Supplementary material for: Effects of mind-body exercise in chronic cardiopulmonary dyspnoea patients—a network meta-analysis of randomized controlled trials
Source: Front Cardiovasc Med. 2025 Jun 4;12:1546996. doi: 10.3389/fcvm.2025.1546996 (PMC12174109; doi:10.3389/fcvm.2025.1546996)
Supplement: Supplementary file 3 [file Table3.docx]

**Table S3 THE ST.GEORGE'S HOSPITAL RESPIRATORY QUESTIONNAIRE (SGRQ)**

*This questionnaire is designed to help us learn much more about how your*

*breathing is troubling you and how it affects your life. We are using it to find out*

*which aspects of your illness cause you most problems,rather than what the*

*doctors and nurses think your problems are.*

*Please read the instructions carefully and ask questions if you do not understand anything.Do*

*not spend too long deciding on your answers.*

*Before completing the rest of the questionnaire:*

*Please check mark one box to show how you describe* Very good Good Fair Poor Very poor

*your present health:*

□ □ □ □ □

**Table S3 St.George's Respiratory Questionnaire**

**PART 1**

| **Questions** **about** **how** **much** **chest** **problem** **you** **have** **had** **over** **the** **past** **4** **weeks.** | | | | | |
| --- | --- | --- | --- | --- | --- |
| Please checkmark( √)one box for each question | | | | | |
|  | Most days a week | Several days a week | A few days a month | Only with chest infections | Not  at all |
| 1. Over the past 4 weeks,I have coughed: | **□** | **□** | **□** | **□** | **□** |
| 2. Over the past 4 weeks, I have brought up phlegm (sputum): | **□** | **□** | **□** | **□** | **□** |
| 3. Over the past 4 weeks, I have had shortness of breath: | **□** | **□** | **□** | **□** | **□** |
| 4. Over the past 4 weeks,I have had attacks of wheezing: | **□** | **□** | **□** | **□** | **□** |
| 5. During the past 4 weeks,how many severe or very unpleasant attacks of chest problem have you had? |  | | | | |
|  | Please checkmark (√) one box only: | | | | |
|  | more than 3 attacks: | | | | **□** |
|  | 3 attacks | | | | **□** |
|  | 2 attacks | | | | **□** |
|  | 1 attacks | | | | **□** |
|  | no attacks | | | | **□** |
| 6. How long did the worst attack of chest problem last:(Go to question 7 if you had no severe attacks) |  | | | | |
|  | Please checkmark( √)one box only: | | | | |
|  | a week or more | | | | **□** |
|  | 3 days or more | | | | **□** |
|  | 1 or 2 days | | | | **□** |
|  | Less than a day | | | | **□** |
| 7. Over the past 4 weeks,in an average week,how many good days (with little chest problem)have you had: |  | | | | |
|  | Please checkmark( √)one box only: | | | | |
|  | No good days | | | | **□** |
|  | 1 or 2 good days | | | | **□** |
|  | 3 or 4 good days | | | | **□** |
|  | Nearly every day was good | | | | **□** |
|  | Every day was good | | | | **□** |
| 8. If you have a wheeze,is it worse in the moming: |  | | | | |
|  | Please checkmark( √)one box only: | | | | |
|  | No | | | | **□** |
|  | Yes | | | | **□** |

**Table S3 St.George's Respiratory Questionnaire**

**PART2**

| **Section 1** | | | | |
| --- | --- | --- | --- | --- |
| How would you describe your chest condition? | | | | |
| Please checkmark (√) one box only. | | | | |
| The most important problem I have | | | **□** |  |
| Causes me quite a lot of problems | | | **□** |  |
| Causes me a few problems | | | **□** |  |
| Causes me no problem | | | **□** |  |
| If you have ever had paid employment. |  | | | |
|  | Please checkmark (√) one box only: | | | |
| My chest problem made me stop work altogether | | | | **□** |
| My chest problem interferes with my work or made me change my work | | | | **□** |
| My chest problem does not affect my work | | | | **□** |
| **Section 2** |  | | |  |
| ***Questions about what activities usually make you feel breathless these days****.* | | | |  |
| **For each item, please checkmark (√) the box as it applies to you these days:** | | | | |
|  | True | False | | |
| Sitting or lying still | **□** | **□** | | |
| Getting washed or dressed | **□** | **□** | | |
| Walking around at home | **□** | **□** | | |
| Walking outside on the level | **□** | **□** | | |
| Climbing up a flight of stairs | **□** | **□** | | |
| Climbing hills | **□** | **□** | | |
| Playing sports or games | **□** | **□** | | |
|  |  | | | |

**Table S3 St.George's Respiratory Questionnaire**

**PART2**

| **Section 3** | | | |
| --- | --- | --- | --- |
| ***Some more questions about your cough and breathlessness these days.*** | | | |
| **For each item, please checkmark (√) the box as it applies to you these days:** | | | |
|  | True | False | |
| My cough hurts | **□** | **□** | |
| My cough makes me tired | **□** | **□** | |
| l am breathless when I talk | **□** | **□** | |
| l am breathless when I bend over | **□** | **□** | |
| My cough or breathing disturbs my sleep | **□** | **□** | |
| I get exhausted easily | **□** | **□** | |
|  |  |  | |
| **Section 4** |  |  | |
| ***Questions about other effects that your chest problem may have on you these days.*** | | | |
| **For each item, please check mark (√) the box as it applies to you these days:** | | | |
|  | | True | False |
| My cough or breathing is embarrassing in public | | **□** | **□** |
| My chest problem is a nuisance to my family,friends or neighbours | | **□** | **□** |
| l get afraid or panic when I cannot get my breath | | **□** | **□** |
| l feel that I am not in control of my chest problem | | **□** | **□** |
| I do not expect my chest to get any better | | **□** | **□** |
| I have become frail or an invalid because of my chest | | **□** | **□** |
| Exercise is not safe for me | | **□** | **□** |
| Everything seems too much of an effort | | **□** | **□** |
|  |  |  | |
| **Section 5** |  |  | |
| ***Questions about your medication.If you are taking no medication go straight to Section 6.*** | | | |
| **For each item, please checkmark (√) the box as it applies to you these days:** | | | |
|  | True | False | |
| My medication does not help me very much | **□** | **□** | |
| I get embarrassed using my medication in public | **□** | **□** | |
| I have unpleasant side effects from my medication | **□** | **□** | |
| My medication interferes with my life a lot | **□** | **□** | |
|  |  |  | |

**Table S3 St.George's Respiratory Questionnaire**

**PART2**

| **Section 6** | | | |
| --- | --- | --- | --- |
| ***These are questions about how your activities might be affected by your breathing*** | | | |
| **For each item, please checkmark(√) the box as it applies to you because of your breathing:** | | | |
|  | | True | False |
| l take a long time to get was hed or dressed | | **□** | **□** |
| I cannot take a bath or shower,or I take a long time | | **□** | **□** |
| I walk slower than other people,or I stop for rests | | **□** | **□** |
| Jobs such as housework take a long time,or I have to stop for rests | | **□** | **□** |
| If I walk up one flight of stairs,I have to go slowly or stop | | **□** | **□** |
| If I hurry or walk fast,I have to stop or slow down | | **□** | **□** |
| My breathing makes it difficult to do things such as climbing up hills,carrying things up stairs,light gardening such as weeding,dancing,playing bowls or golf | | **□** | **□** |
| My breathing makes it difficult to do things such as carrying heay loads, digging the garden or shovelling snow,jogging or walking at 8 kilometres per hour,playing tennis or swimming | | **□** | **□** |
| My breathing makes it difficult to do things such as very heavy manual work, running,cycling,swimming fast or playing competitive sports | | **□** | **□** |
| **Section 7** |  |  | |
| ***We would like to know how your chest problem usually affects your daily life*** | | | |
| **For each item, please checkmark(√) the box as it applies to you because of your chest problem:** | | | |
|  | True | False | |
| I cannot play sports or games | **□** | **□** | |
| I cannot go out for entertainment or recreation | **□** | **□** | |
| I cannot go out of the house to do the groceries | **□** | **□** | |
| I cannot do housework | **□** | **□** | |
| I cannot move far from my bed or chair | **□** | **□** | |
|  |  |  | |

**Table S3 St.George's Respiratory Questionnaire**

| ***Here is a list of other activities that your chest problem may prevent you doing(you do not have to check mark these,they are just to remind you of ways in which your breathlessness may affect you):*** | |
| --- | --- |
| Going for walks or walking the dog | |
| Doing things at home or in the garden | |
| Sexual intercourse | |
| Going out to church or place of entertainment | |
| Going out in bad weather or into smoky rooms | |
| Visiting family or friends or playing with children | |
|  | |
| ***Please write in any other important activities that your chest problem may stop you doing:*** | |
|  | |
|  | |
|  | |
|  | |
|  | |
| ***Now,would you check mark the box(one only)which you think best describes how your chest affects you:*** | |
| It does not stop me doing anything I would like to do | **□** |
| It stops me doing one or two things I would like to do | **□** |
| It stops me doing most of the things I would like to do | **□** |
| It stops me doing everything I would like to do | **□** |
| Thank you for flling in this questionnaire.Before you finish,would you check to see that you have answered all the questions. | |
|  | |
