# Supplementary material for: Effects of mind-body exercise in chronic cardiopulmonary dyspnoea patients—a network meta-analysis of randomized controlled trials
Source: Front Cardiovasc Med. 2025 Jun 4;12:1546996. doi: 10.3389/fcvm.2025.1546996 (PMC12174109; doi:10.3389/fcvm.2025.1546996)
Supplement: Supplementary file 4 [file Table4.docx]

**Table S4 CONTENT AND STRUCTURE OF THE FINAL CAT QUESTIONNAIRE**

**How is your COPD?**

For each item below,place a mark( √)in the box that best describes your experience

l am very sad

| 0 | 1 | 2 | 3 | 4 | 5 |
| --- | --- | --- | --- | --- | --- |

**Example:** I am very happy

**SCORE**

| I never cough | \| 0 \| 1 \| 2 \| 3 \| 4 \| 5 \| \| --- \| --- \| --- \| --- \| --- \| --- \| | I cough all the time |  |  |  |  |
| --- | --- | --- | --- | --- | --- | --- | --- | --- | --- | --- | --- | --- |
| I have no phlegm (mucus) in my chest at all | \| 0 \| 1 \| 2 \| 3 \| 4 \| 5 \| \| --- \| --- \| --- \| --- \| --- \| --- \| | My chest is completely full of phlegm(mucus) |  |  |  |  |
| My chest does not feel tight at all | \| 0 \| 1 \| 2 \| 3 \| 4 \| 5 \| \| --- \| --- \| --- \| --- \| --- \| --- \| | My chest feels very tight |  |  |  |  |
| When I walk up a hill or one flight of stairs l am not  breathless | \| 0 \| 1 \| 2 \| 3 \| 4 \| 5 \| \| --- \| --- \| --- \| --- \| --- \| --- \| | When I walk up a hill or one flight of stairs I am very breathless |  |  |  |  |
| l am not limited doing any activities at home | \| 0 \| 1 \| 2 \| 3 \| 4 \| 5 \| \| --- \| --- \| --- \| --- \| --- \| --- \| | l am not limited doing activities at home |  |  |  |  |
| l am confident leaving my home despite my lung condition | \| 0 \| 1 \| 2 \| 3 \| 4 \| 5 \| \| --- \| --- \| --- \| --- \| --- \| --- \| | l am not at all confident leaving my home because of my lung condition |  |  |  |  |
| I sleep soundly | \| 0 \| 1 \| 2 \| 3 \| 4 \| 5 \| \| --- \| --- \| --- \| --- \| --- \| --- \| | l don't sleep soundly because of my lung condition |  |  |  |  |
| I have lots of energy | \| 0 \| 1 \| 2 \| 3 \| 4 \| 5 \| \| --- \| --- \| --- \| --- \| --- \| --- \| | I have no energy at all |  |  |  |  |

**SCORE**
