# Supplementary material for: Effects of mind-body exercise in chronic cardiopulmonary dyspnoea patients—a network meta-analysis of randomized controlled trials
Source: Front Cardiovasc Med. 2025 Jun 4;12:1546996. doi: 10.3389/fcvm.2025.1546996 (PMC12174109; doi:10.3389/fcvm.2025.1546996)
Supplement: Supplementary file 5 [file Table5.docx]

**Supplementary Table S5.** Risk of bias for each included studies.

| Author | Year | Random  sequence generation | Allocation concealment | Blinding of participants and personnel | Blinding of outcome assessment | Incomplete outcome data | Selective reporting | Other bias | Overall Score (Category) |
| --- | --- | --- | --- | --- | --- | --- | --- | --- | --- |
| L Zheng | 2017 | Low | Unclear | Unclear | High | Low | Low | Low | 3（moderate） |
| Z Zhu | 2010 | Low | Unclear | Unclear | High | Low | Low | Unclear | 3（moderate） |
| M Xu | 2019 | Unclear | Unclear | Unclear | High | Unclear | Low | Unclear | 6（high） |
| H Ewa | 2017 | Low | Unclear | Unclear | High | Low | Low | Low | 3（moderate） |
| GY Yeh | 2013 | Low | Unclear | Unclear | Low | Low | Low | Low | 2（low） |
| LS Redwine | 2019 | Low | Low | Unclear | Low | Low | Low | Low | 1（low） |
| C Giuseppe | 2011 | Low | Low | Unclear | High | Low | Low | Low | 2（low） |
| C Huang | 2014 | Low | Unclear | Unclear | High | Unclear | Low | Low | 4（moderate） |
| ML Yu | 2018 | Low | Low | Unclear | High | Low | Low | Low | 2（low） |
| MI Polkey | 2018 | Unclear | Unclear | Unclear | High | Low | Low | Low | 4（moderate） |
| AWK Chan | 2013 | Low | Unclear | Unclear | High | Low | Low | Unclear | 4（moderate） |
| ST Du | 2012 | Low | Unclear | Unclear | High | Unclear | Low | Low | 4（moderate） |
| ZF Ren | 2017 | Low | Low | Unclear | High | Low | Low | Low | 2（low） |
| Y Zhang | 2019 | Low | Unclear | Unclear | High | Unclear | Low | Low | 4（moderate） |
| XC Zhang | 2014 | Low | Unclear | Unclear | High | Unclear | Low | Low | 4（moderate） |
| M Ding | 2015 | Low | Unclear | Unclear | High | Low | Low | Low | 3（moderate） |
| Y Zhu | 2010 | Low | Unclear | Unclear | High | Low | Low | Low | 3（moderate） |
| XS Dong | 2021 | Low | Unclear | Unclear | Low | Low | Low | Low | 2（low） |
| J Zhang | 2017 | Unclear | Unclear | Unclear | High | Unclear | Low | Low | 5（high） |
| DP fang | 2012 | Low | Unclear | Unclear | Low | Low | Low | Low | 2（low） |
| JX Chen | 2009 | Low | Unclear | Unclear | High | Unclear | Low | Low | 4（moderate） |
| Q Chen | 2021 | Low | Unclear | Unclear | High | Low | Low | Unclear | 4（moderate） |
| CM Xiao | 2015 | Unclear | Unclear | Unclear | Low | Low | Low | Low | 3（moderate） |
| XD Liu | 2012 | Low | Unclear | Unclear | Low | Low | Low | Low | 2（low） |
| Q Li | 2012 | Low | Unclear | Unclear | High | Low | Low | Unclear | 4（moderate） |
| SM Chen | 2020 | Low | Unclear | Unclear | High | Low | Low | Low | 3（moderate） |
| YC Zheng | 2019 | Low | Low | Unclear | High | Low | Low | Low | 2（low） |
| DF Hou | 2017 | Unclear | Unclear | Unclear | High | Low | Low | Low | 4（moderate） |
| G Yin | 2013 | Low | Low | Unclear | High | Unclear | Low | Low | 3（moderate） |
| Y Gao | 2015 | Low | Unclear | Unclear | High | Low | Low | Low | 3（moderate） |
| BHP Ng | 2011 | Low | Low | Unclear | Low | Low | Low | Low | 1（low） |
| L Ng | 2014 | Low | Low | Unclear | Low | Low | Low | Low | 1（low） |
| HJ Liu | 2021 | Low | Unclear | Unclear | High | Unclear | Low | Unclear | 5（high） |
| M Zhou | 2021 | Low | Unclear | Unclear | High | Low | Low | Low | 3（moderate） |
| Jain AK | 2022 | Low | Low | High | Low | Low | Low | Low | 1（low） |
| Yeh GY | 2011 | Low | Unclear | Unclear | Low | Low | Low | Low | 2（low） |
| Yeh GY | 2008 | Low | Low | High | Low | Low | Low | Low | 1（low） |
| Donesky D | 2017 | Unclear | Unclear | High | Unclear | Low | Low | Unclear | 5（high） |
| Pullen PR | 2008 | Low | Unclear | High | Low | Low | Low | Low | 2（low） |
| Yeh GY | 2004 | Low | Low | High | Low | Low | Low | Low | 1（low） |
| Ma CH | 2022 | Low | Low | Unclear | Low | Low | Low | Low | 1（low） |
| Chen XK | 2021 | Low | Low | Unclear | Low | Low | Low | Low | 1（low） |
| Krishna BH | 2014 | Unclear | Unclear | High | Low | Low | Low | Unclear | 4（moderate） |
| Srisoongnern S | 2021 | Low | Low | Unclear | Low | Low | Low | Low | 1（low） |
