# Supplementary material for: Effects of mind-body exercise in chronic cardiopulmonary dyspnoea patients—a network meta-analysis of randomized controlled trials
Source: Front Cardiovasc Med. 2025 Jun 4;12:1546996. doi: 10.3389/fcvm.2025.1546996 (PMC12174109; doi:10.3389/fcvm.2025.1546996)
Supplement: Supplementary file 6 [file Table6.docx]

**Supplementary Table S6.** Consistency test for MLHFQ.

|  | Coef. | Std. Err. | z | P>\|z\| | [95% Conf. Interval] |  |
| --- | --- | --- | --- | --- | --- | --- |
| B VS CON | 7.037347 | 12.02443 | 0.59 | 0.558 | -16.5301 | 30.6048 |
| C VS CON | 12.84223 | 10.67389 | 1.2 | 0.229 | -8.078211 | 33.76266 |
| D VS CON | 1.917287 | 14.32128 | 0.13 | 0.894 | -26.15191 | 29.98649 |
| E VS CON | 0.6516956 | 11.78731 | 0.06 | 0.956 | -22.45101 | 23.7544 |
| F VS CON | -1.135618 | 12.12545 | -0.09 | 0.925 | -24.90106 | 22.62983 |
