# Supplementary material for: Effects of mind-body exercise in chronic cardiopulmonary dyspnoea patients—a network meta-analysis of randomized controlled trials
Source: Front Cardiovasc Med. 2025 Jun 4;12:1546996. doi: 10.3389/fcvm.2025.1546996 (PMC12174109; doi:10.3389/fcvm.2025.1546996)
Supplement: Supplementary file 7 [file Table7.docx]

**Supplementary Table S7.** Consistency test for 6MWD.

|  | Coef. | Std. Err. | z | P>\|z\| | [95% Conf. Interval] |  |
| --- | --- | --- | --- | --- | --- | --- |
| B VS CON | 47.77246 | 34.41435 | 1.39 | 0.165 | -19.67843 | 115.2234 |
| C VS CON | 16.9367 | 32.94171 | 0.51 | 0.607 | -47.62787 | 81.50128 |
| D VS CON | 51.96194 | 35.84256 | 1.45 | 0.147 | -18.28818 | 122.2121 |
| E VS CON | 33.3421 | 35.6107 | 0.94 | 0.349 | -36.45358 | 103.1378 |
| F VS CON | 35.48863 | 33.88678 | 1.05 | 0.295 | -30.92824 | 101.9055 |
| G VS CON | 7.580524 | 44.284 | 0.17 | 0.864 | -79.21452 | 94.37557 |
| H VS CON | 38.78051 | 43.8651 | 0.88 | 0.377 | -47.19352 | 124.7545 |
| I VS CON | 118.0406 | 39.11782 | 3.02 | 0.003 | 41.37109 | 194.7101 |
