# Supplementary material for: Effects of mind-body exercise in chronic cardiopulmonary dyspnoea patients—a network meta-analysis of randomized controlled trials
Source: Front Cardiovasc Med. 2025 Jun 4;12:1546996. doi: 10.3389/fcvm.2025.1546996 (PMC12174109; doi:10.3389/fcvm.2025.1546996)
Supplement: Supplementary file 8 [file Table8.docx]

**Supplementary Table S8.** Consistency test for SGRQ.

|  | Coef. | Std. Err. | z | P>\|z\| | [95% Conf. Interval] |  |
| --- | --- | --- | --- | --- | --- | --- |
| B VS CON | 0.1684967 | 3.027736 | 0.06 | 0.956 | -5.765757 | 6.10275 |
| C VS CON | -2.505626 | 3.947539 | -0.63 | 0.526 | -10.24266 | 5.231408 |
| D VS CON | -7.515611 | 3.819789 | -1.97 | 0.049 | -15.00226 | -0.0289622 |
| E VS CON | -2.149621 | 3.35102 | -0.64 | 0.521 | -8.717499 | 4.418258 |
