# Supplementary material for: Effects of mind-body exercise in chronic cardiopulmonary dyspnoea patients—a network meta-analysis of randomized controlled trials
Source: Front Cardiovasc Med. 2025 Jun 4;12:1546996. doi: 10.3389/fcvm.2025.1546996 (PMC12174109; doi:10.3389/fcvm.2025.1546996)
Supplement: Supplementary file 9 [file Table9.docx]

**Supplementary Table S9.** Consistency test for CAT.

|  | Coef. | Std. Err. | z | P>\|z\| | [95% Conf. Interval] |  |
| --- | --- | --- | --- | --- | --- | --- |
| B VS CON | 4.779143 | 1.592837 | 3 | 0.003 | 1.65724 | 7.901046 |
| C VS CON | 4.951109 | 2.509456 | 1.97 | 0.048 | 0.0326651 | 9.869553 |
| D VS CON | 1.292101 | 2.070777 | 0.62 | 0.533 | -2.766548 | 5.350749 |
| E VS CON | 2.378995 | 3.168848 | 0.75 | 0.453 | -3.831832 | 8.589822 |
