# Supplementary material for: Effects of mind-body exercise in chronic cardiopulmonary dyspnoea patients—a network meta-analysis of randomized controlled trials
Source: Front Cardiovasc Med. 2025 Jun 4;12:1546996. doi: 10.3389/fcvm.2025.1546996 (PMC12174109; doi:10.3389/fcvm.2025.1546996)
Supplement: Supplementary file 10 [file Table10.docx]

**Supplementary Table S10.** Consistency test for NT-proBNP.

|  | Coef. | Std. Err. | z | P>\|z\| | [95% Conf. Interval] |  |
| --- | --- | --- | --- | --- | --- | --- |
| B VS CON | 37.48187 | 122.5799 | 0.31 | 0.76 | -202.7704 | 277.7341 |
| C VS CON | -203.2181 | 542.7705 | -0.37 | 0.708 | -1267.029 | 860.5925 |
| D VS CON | 25.48191 | 122.724 | 0.21 | 0.836 | -215.0528 | 266.0166 |
| E VS CON | -148.804 | 126.6496 | -1.17 | 0.24 | -397.0327 | 99.42462 |
