# Supplementary figures and images for: Effects of mind-body exercise in chronic cardiopulmonary dyspnoea patients—a network meta-analysis of randomized controlled trials
Source: Front Cardiovasc Med. 2025 Jun 4;12:1546996. doi: 10.3389/fcvm.2025.1546996 (PMC12174109; doi:10.3389/fcvm.2025.1546996)

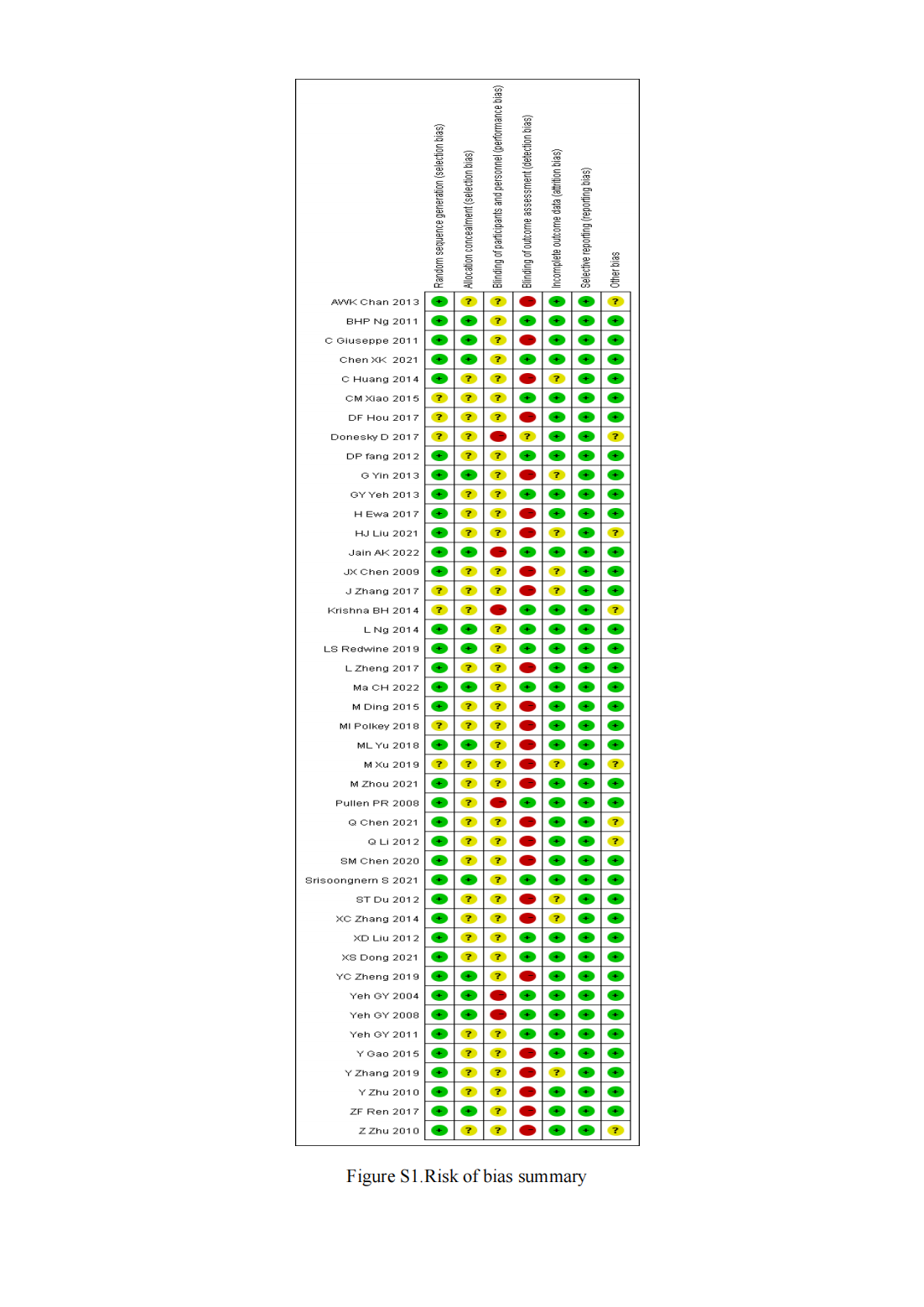

Supplement: Supplementary file 11 [file Image1.tif]

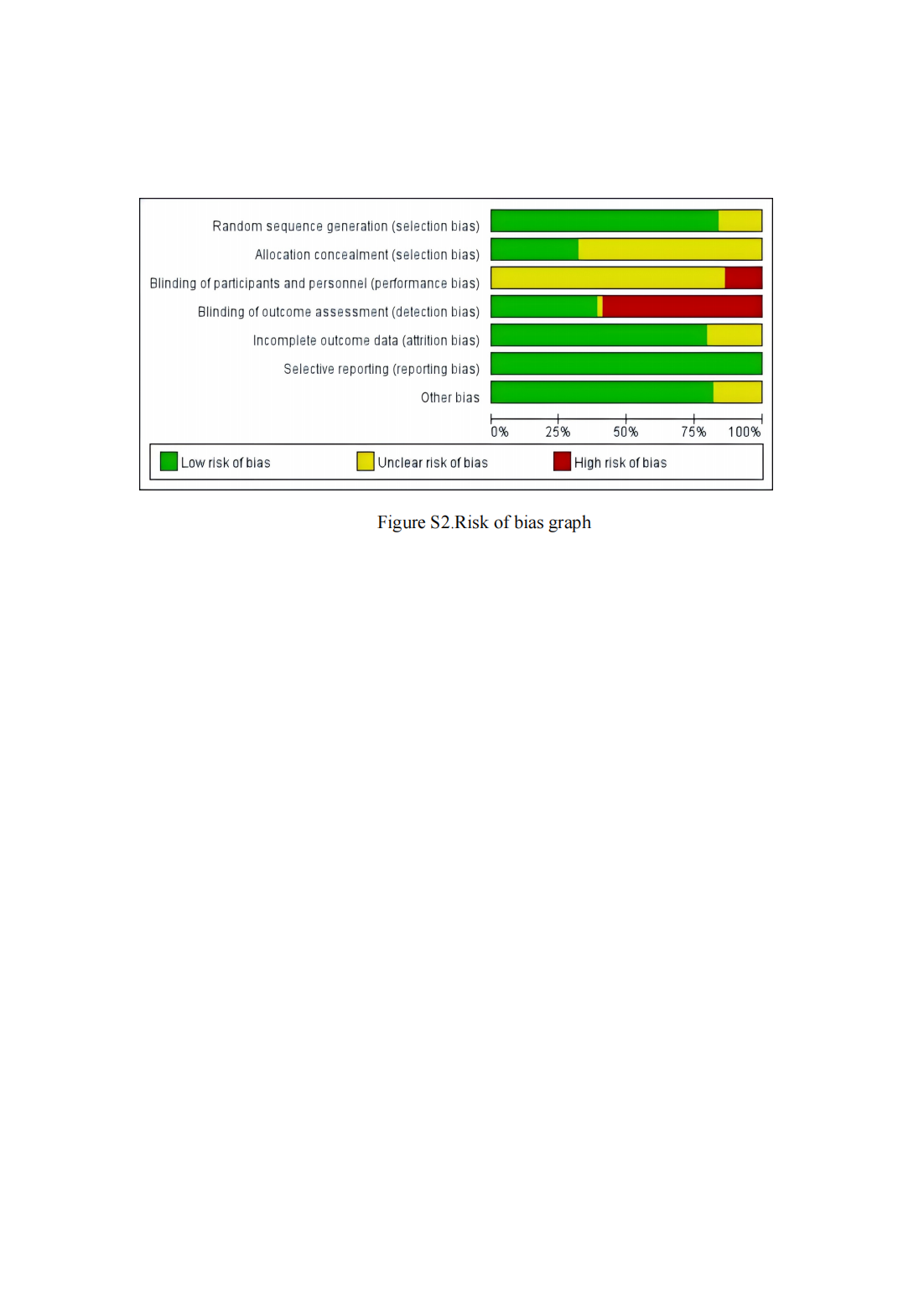

Supplement: Supplementary file 12 [file Image2.tif]

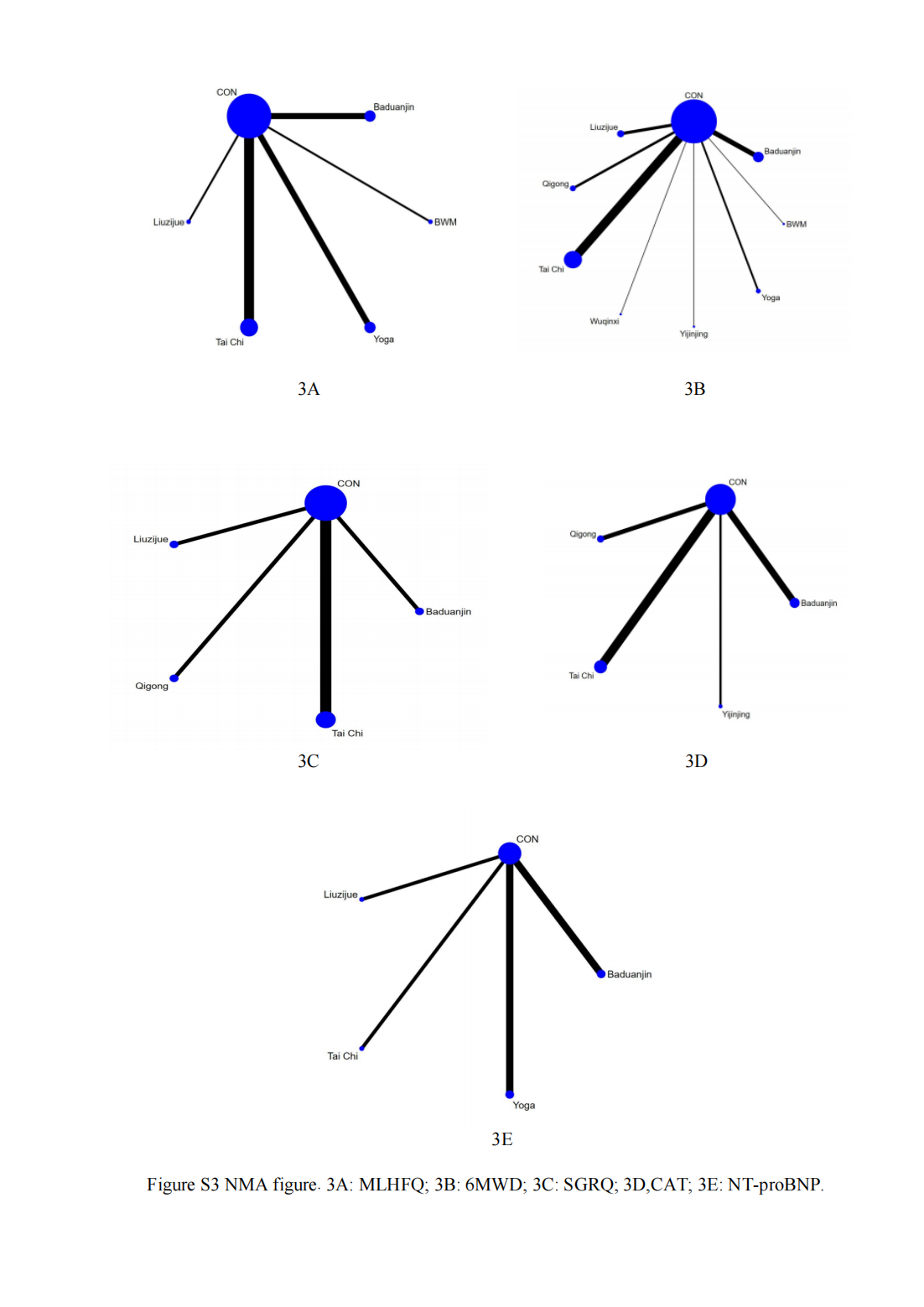

Supplement: Supplementary file 13 [file Image3.tif]
